# Haemoglobin causes neuronal damage *in vivo* which is preventable by haptoglobin

**DOI:** 10.1093/braincomms/fcz053

**Published:** 2020-01-03

**Authors:** Patrick Garland, Matthew J Morton, William Haskins, Ardalan Zolnourian, Andrew Durnford, Ben Gaastra, Jamie Toombs, Amanda J Heslegrave, John More, Azubuike I Okemefuna, Jessica L Teeling, Jonas H Graversen, Henrik Zetterberg, Soren K Moestrup, Diederik O Bulters, Ian Galea

**Affiliations:** 1 Clinical Neurosciences, Clinical and Experimental Sciences, Faculty of Medicine, University of Southampton, Southampton, SO16 6YD, UK; 2 Department of Neurosurgery, Wessex Neurological Centre, University Hospital Southampton NHS Foundation Trust, Southampton, SO16 6YD, UK; 3 UK Dementia Research Institute, University College London, London, WC1E 6BT, UK; 4 Department of Neurodegenerative Disease, Institute of Neurology, London, WC1N 3BG, UK; 5 Research & Development Department, Bio Products Laboratory Limited, Elstree, Hertfordshire, WD6 3BX, UK; 6 School of Biological Sciences, Faculty of Environmental and Life Sciences, University of Southampton, Southampton, SO16 6YD, UK; 7 Department of Molecular Medicine, University of Southern Denmark, 5000 Odense C, Denmark; 8 Department of Psychiatry and Neurochemistry, Institute of Neuroscience and Physiology, The Sahlgrenska Academy, University of Gothenburg, Mölndal, S-431 80, Sweden; 9 Clinical Neurochemistry Laboratory, Sahlgrenska University Hospital, Mölndal, S-431 80, Sweden; 10 Department of Clinical Biochemistry, Aarhus University Hospital, 8200 Aarhus N, Denmark; 11 Department of Biomedicine, Aarhus University, 8000 Aarhus C, Denmark

**Keywords:** subarachnoid haemorrhage, haptoglobin, haemoglobin, neurofilament light chain, outcome

## Abstract

After subarachnoid haemorrhage, prolonged exposure to toxic extracellular haemoglobin occurs in the brain. Here, we investigate the role of haemoglobin neurotoxicity *in vivo* and its prevention. In humans after subarachnoid haemorrhage, haemoglobin in cerebrospinal fluid was associated with neurofilament light chain, a marker of neuronal damage. Most haemoglobin was not complexed with haptoglobin, an endogenous haemoglobin scavenger present at very low concentration in the brain. Exogenously added haptoglobin bound most uncomplexed haemoglobin, in the first 2 weeks after human subarachnoid haemorrhage, indicating a wide therapeutic window. In mice, the behavioural, vascular, cellular and molecular changes seen after human subarachnoid haemorrhage were recapitulated by modelling a single aspect of subarachnoid haemorrhage: prolonged intrathecal exposure to haemoglobin. Haemoglobin-induced behavioural deficits and astrocytic, microglial and synaptic changes were attenuated by haptoglobin. Haptoglobin treatment did not attenuate large-vessel vasospasm, yet improved clinical outcome by restricting diffusion of haemoglobin into the parenchyma and reducing small-vessel vasospasm. In summary, haemoglobin toxicity is of clinical importance and preventable by haptoglobin, independent of large-vessel vasospasm.

## Teaser

Extracellular haemoglobin is toxic; its contribution to neuronal damage *in vivo* was unknown. Garland *et al* show that exposing the brain to haemoglobin recapitulates the clinicopathological features of subarachnoid haemorrhage. These features are preventable by administration of haptoglobin, which keeps haemoglobin out of the brain tissue, limiting neuronal damage.

## Graphical Abstract

**Graphical Abstract:**
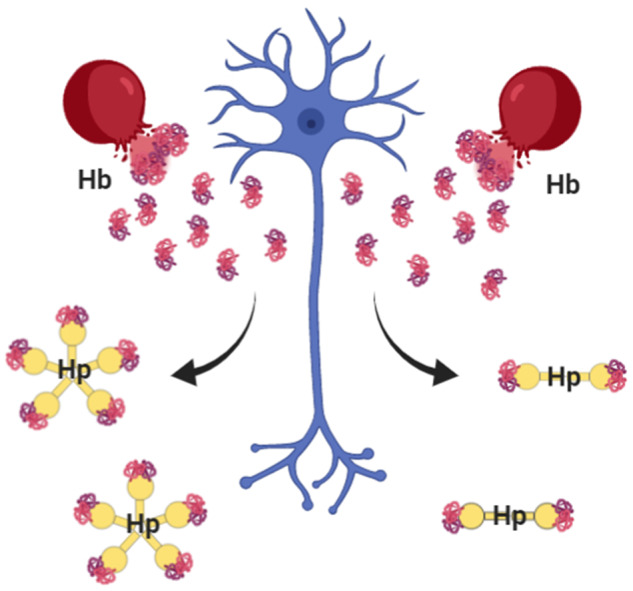

## Introduction

After subarachnoid haemorrhage (SAH), blood is released into the subarachnoid space (Macdonald and Schweizer, 2017). As the blood clot in the subarachnoid space is resorbed, red blood cell lysis leads to the release of cell-free haemoglobin (Hb) over a protracted period exerting a further secondary, and thus potentially reversible, injury. There is a sound biological rationale to hypothesize that prolonged exposure to Hb in the subarachnoid compartment impacts long-term outcome (Bulters *et al.*, 2018). Outside of the controlled environment of the erythrocyte, Hb and its breakdown products (haem, bilirubin and free iron) are involved in toxic redox reactions via the iron atom (Reeder, 2010, 2017), leading to oxidation of DNA, protein and lipids, and hence cellular dysfunction, and brain injury.

Focal intracortical injection of a high concentration of Hb causes seizures (Rosen and Frumin, 1979). Similar injections of whole blood in the striatum of mice cause neurologic deficits such as posturing, incoordination and/or paresis (Zhao *et al.*, 2009). Less is known about the neurological consequences of prolonged exposure to lower concentrations of Hb distributed throughout the CSF space, as happens during clot lysis after SAH. Repeated cisterna magna injections of washed autologous red blood cells in rabbits caused iron deposition and a microglial reaction in the cerebellum, but neuronal integrity and behavioural deficits were not studied (Koeppen and Borke, 1991). A single intraventricular Hb injection in 7-day-old neonatal rats caused neuronal damage (Garton *et al.*, 2016), so a similar process could occur after prolonged exposure to subarachnoid Hb in adults.

Haptoglobin (Hp) is the body’s natural defence against extracellular Hb (Andersen *et al.*, 2017). Hp lowers the redox potential of Hb to prevent damaging peroxidative reactions, and chaperones Hb to prevent haem release and its degradation to free iron. Intrathecal Hp concentration is significantly lower compared with plasma, and most of the Hb after SAH is not bound to Hp (Galea *et al.*, 2012). Therefore, direct supplementation with exogenous Hp, which would be expected to increase CNS Hp concentration more rapidly and to a higher level than drug-induced upregulation of Hp synthesis, might be expected to be of therapeutic benefit after SAH.

This project consists of human, *in vitro* and *in vivo* animal studies, presented in this order. First, we sought to identify whether tissue damage was linked to Hb after SAH in humans. Then, in experimental models, we tested whether prolonged intrathecal exposure to pure Hb is neurotoxic and causes behavioural abnormalities similar to those seen after human SAH. To model prolonged intrathecal Hb exposure, the mouse ventricular system was infused with a clinically relevant Hb concentration for 2 weeks. We studied behavioural deficits, large-vessel and parenchymal vasospasm, the astrocytic and microglial/macrophage reaction to Hb, and synaptic loss. Finally, we assessed if these changes were reversed by administration of Hp.

## Materials and methods

### Human study

Human studies were performed in accordance with the ethical standards as laid down in the 1964 Declaration of Helsinki. Nineteen control participants were patients with non-inflammatory non-haemorrhagic conditions who underwent lumbar puncture and were subsequently found to have normal CSF (National Research Ethics Committee approval number 11/SC/0204). Forty-four Fisher grade III–IV non-traumatic SAH patients, requiring an external ventricular drain (EVD) as part of their clinical management (to manage acute hydrocephalus), were recruited (National Research Ethics Committee approval number 12/SC/0666). CSF was obtained from the EVD on alternate days until removal of the EVD. CSF was drawn from a three-way tap connecting the ventricular catheter (∼30 cm long) to the tubing leading to an external CSF drainage and monitoring system (Becker^®^, Medtronic). For sampling, the tap was opened to the ventricular catheter and closed to the drainage system. The first 3 ml of CSF (representing dead space) was discarded to ensure fresh CSF was obtained. Only 42 patients contributed to this study due to early removal of the EVD. CSF was centrifuged at 1500 rcf for 10 min at 21°C and frozen within 1 h of sampling.

**Figure 1:**
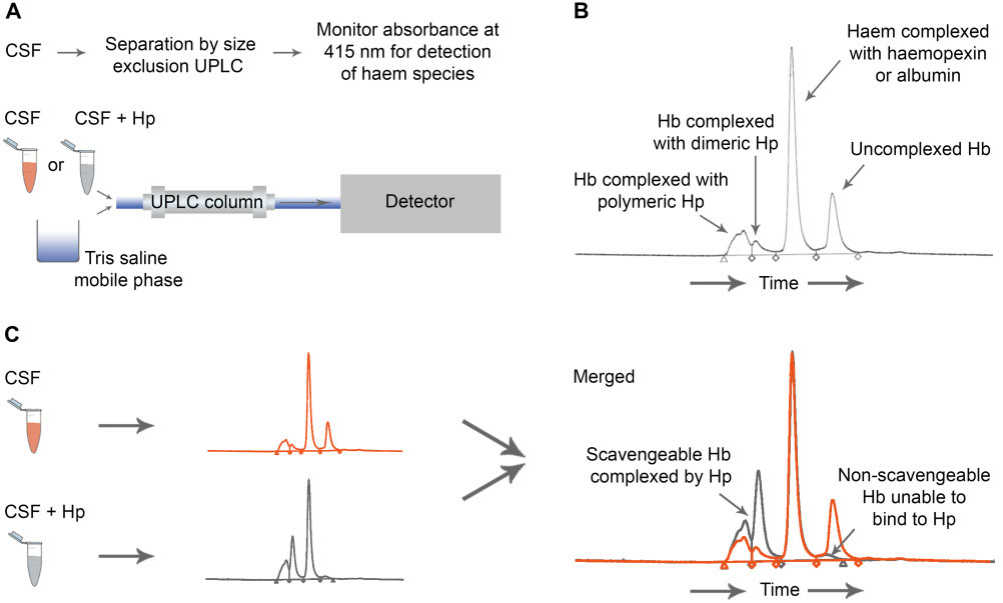
**Human study: methodology.** Assay of Hb binding by Hp. (**A**) UPLC method. CSF alone, or CSF incubated with excess Hp, was run through a C18 column to achieve separation of proteins. The absorbance of the eluate was measured at 415 nm to detect haem-containing species. (**B**) A representative CSF chromatogram demonstrating peaks of Hb complexed to dimeric and polymeric Hp, haem complexed to haemopexin and albumin, and uncomplexed Hb. Peak area under the curve was quantified using a Hb standard curve. (**C**) Uncomplexed Hb able to bind Hp was measured by running CSF alone (orange), and separately CSF to which excess Hp was added (grey). The increase in complexed Hb peak area after adding excess Hp represents uncomplexed Hb that can bind Hp. The uncomplexed Hb peak remaining after adding excess Hp represents uncomplexed Hb unable to bind Hp.

Ultra-performance liquid chromatography (UPLC) was used to separate CSF components in a tris-saline mobile phase, coupled to absorbance measurement at 415 nm to identify haem-containing species. Full details are in Supplementary material. Hb was measured in CSF before and after saturating with Hp (Bio Products Laboratory Limited, Elstree, UK) to measure Hp-bindable and Hp-unbindable uncomplexed Hb (Fig. 1). CSF neurofilament light chain (NFL) was measured by enzyme-linked immunosorbent assay (UmanDiagnostics, Umea, Sweden; Supplementary material).

### *In vitro* study

Primary hippocampal neurones were cultured according to a modified method from the Ittner lab (Fath *et al.*, 2009; Garland *et al.*, 2012; Supplementary material). Cultured neurones were challenged with Hb at day 7 until day 14. Over the 7 days, Hb concentration was maintained during media changes. An equimolar-binding ratio of Hp (see Supplementary material) was added concomitantly for Hp treatment experiments. Hb and Hp preparations, immunocytochemistry for microtubule-associated protein-2 and quantification of viable neurones are detailed in Supplementary material.

### *In vivo* animal model

#### Mouse model of prolonged intrathecal Hb exposure

To model prolonged intrathecal Hb exposure, a 2-week intracerebroventricular infusion of Hb was used. Locally bred male C57BL/6 mice were housed at 21°C under a 12-h light/12-h dark cycle in a conventional animal research facility and allowed access to food and water *ad libitum*; surgery was performed at 10–12 weeks of age. All procedures were performed under UK Home Office licence 30-3057 and all applicable international, national and/or institutional guidelines for the care and use of animals were followed. Alzet osmotic minipumps (Model 2002, #296, 2-week infusion, 0.5 µl/h, Charles River, Harlow, Essex) were loaded with vehicle (0.9% saline), Hb, Hb + Hp or Hp. Treatment allocation was random. The infusate Hb concentration was 20 mg/ml to achieve a clinically relevant CSF Hb concentration of 10 µM as detailed in Supplementary material. The Hp concentration was 14 mg/ml to achieve 1:1 Hb to Hp binding stoichiometry, guided by data from empirical determination of Hb to Hp binding ratio (Supplementary material). The minipump was attached to a 30-gauge ventricular cannula using vinyl tubing (Brain Infusion Kit 3, #8851, Charles River, Harlow, Essex) and primed with test solution according to the manufacturer’s instructions. Mice were anaesthetized using a ketamine (Bayer, Reading, UK) and xylazine (Pfizer, Tadworth, Surrey, UK) mixture (100 and 10 mg/kg, respectively). The head was immobilized in a stereotactic frame. Mice body temperature was thermostatically controlled with a rectal probe and a heated pad. An aseptic technique was used. Blunt dissection was used to create a subcutaneous pocket in the interscapular region for insertion of the minipump. A burr hole was drilled and the ventricular cannula inserted at the following coordinates from bregma: anteroposterior, −0.4 mm; lateral, 1 mm; depth, 2.5 mm. Cyanoacrylate was used to secure the cannula holder to the skull surface. Buprenorphine (Centaur, Castle Cary, Somerset, UK) was administered immediately following surgery (0.5 mg/kg subcutaneously) and the next morning in 5 ml fruit jelly (300 μg/45 ml). Mice were housed individually following surgery for 14 days so the experimental unit was a single animal. Mice were checked twice in the first 24 h and daily thereafter. Residual volume in the minipumps was systematically examined after explantation, at the end of the 2 weeks, to ensure the appropriate volume of 200 µl was delivered while *in situ*. Correct placement of the ventricular cannula was confirmed during tissue sectioning by observing the cannula track.

#### Behavioural, histological and biochemical readouts

Open-field behavioural analysis was performed in a laser-monitored behaviour arena (Med Associates, Fairfax, VA, USA) over a 5-min period, twice a day at the same time in the morning and afternoon, as detailed in Supplementary material. At 14 days post-surgery, mice were transcardially perfused with heparinized saline (1 unit/ml, LEO Pharma, Hurley, Berkshire, UK), followed by 4% paraformaldehyde in 0.1 M phosphate buffer. Brains were removed and post-fixed in 4% paraformaldehyde overnight at 4°C and then embedded in paraffin wax. Coronal sections were cut at 10 µm, dewaxed in xylene and rehydrated through graded ethanol/water solutions. Immunohistochemistry and imaging for glial fibrillary acidic protein (GFAP), ionized calcium-binding adapter molecule 1 (Iba1), synaptophysin, Hb, CD163 and smooth-muscle actin (SMA), and Perl’s histochemistry was performed as detailed in Supplementary material. To ensure blinding, all samples were coded prior to image capture and analysis. All analyses were performed by two-dimensional quantification, averaged over two sections per animal within each area studied, and data are presented per mm^2^, unless otherwise indicated; further details are in Supplementary material. For biochemistry, the hemisphere ipsilateral to cannula insertion was homogenized in buffer, after transcardial perfusion with heparinized saline only and tissue processed for Western blotting as detailed in Supplementary material.

#### Fluorescent imaging of Circle of Willis *in situ*

The method for *in situ* imaging was established in the Zipfel group (Aum *et al.*, 2017). Mice were anaesthetized using a ketamine (Bayer, Reading, UK) and xylazine (Pfizer, Tadworth, Surrey, UK) mixture (100 and 10 mg/kg, respectively) and transcardially perfused through the left ventricle with 10 mM glucose–physiological-buffered saline (PBS) followed by 20 ml 20 μM 5-(6)-carboxy-X-rhodamine, succinimidyl ester (Sigma-Aldrich, Gillingham, Dorset, UK) dye in 10 mM glucose-PBS prior to perfusions with 4% paraformaldehyde (in PBS). All perfusions were performed with solutions at 21°C, at a constant pressure of 80 ± 2 mmHg using a GE Druck DP1705. Animals were decapitated, the calvaria removed and post-fixed in 4% paraformaldehyde in the dark at 4°C for 24 h. Brains were removed under a dissection microscope to preserve the basal arteries. Then, brains were placed *en bloc* on a glass coverslip and gently covered in PBS before placing on the stage of a confocal laser scanning microscope (SP8, Leica, Wetzlar, Germany). Measurements of anterior and middle cerebral artery (MCA) diameters were made at the narrowest point across the first millimetre of the vessel, as detailed in Supplementary material.

### Surface plasmon resonance

Surface plasmon resonance analysis was carried out as described (Madsen *et al.*, 2004) using a Biacore 3000 instrument (Biacore, Uppsala, Sweden). Mouse or human CD163, produced using recombinant technology in-house (Kristiansen *et al.*, 2001; Etzerodt *et al.*, 2013), was immobilized in 10 mM sodium acetate (pH 4) and remaining binding sites were blocked with 1 M ethanolamine (pH 8.5). The resulting densities were: human CD163 0.0468 pmol/mm^2^ and murine CD163 0.0320 pmol/mm^2^. Sensorgrams were generated using the running buffer CaHBS with 2 mM free Ca_2_Cl_2_ (10 mM Hepes, 150 mM NaCl, 3.0 mM CaCl_2_, 1.0 mM EGTA + 0.005% P20, and pH 7.4) and the protein concentrations: 5 µg/ml of mouse and human Hb (Sigma-Aldrich, Gillingham, Dorset, UK); 7.5 µg/ml of mouse Hp (MyBioSource, San Diego, Ca) and human Hp1-1 (Sigma-Aldrich, Gillingham, Dorset, UK). The flow cell was regenerated in 10 mM glycine, 20 mM EDTA, 500 mM NaCl + 0.005% P20, pH4.0 between the runs. All experiments were conducted at least in triplicate and data was evaluated using the BiaEvaluation ver. 4.1 software (Biacore, Uppsala, Sweden).

### Statistical analysis

Statistical analysis and graph preparation were performed using SPSS (v24) and GraphPad Prism (v7.01), with data expressed as mean ± standard error of the mean (SEM), median ± inter-quartile range or 95% confidence intervals. Normality and heteroscedasticity were routinely determined across all data sets. Where necessary, logarithmic transformation was used to normalize data. Alpha (α), the probability of a Type I error, was 0.05. Two-tailed hypotheses were considered throughout. Details of individual statistical analyses are available in Supplementary material. Animals were randomly selected and sequentially assigned to treatment groups. Animal *in vivo* experiments were reported according to ARRIVE guidelines (Kilkenny *et al.*, 2010). Supplementary Table 1 summarizes all the analyses performed and the results.

### Data availability

Datasets are available from the corresponding author on reasonable request in accordance with the University of Southampton’s data-sharing policies, ethical approvals and contracts with the co-authors and their institutions.

## Results

### Human study

#### NFL concentration in CSF is predicted by Hb

**Figure 2:**
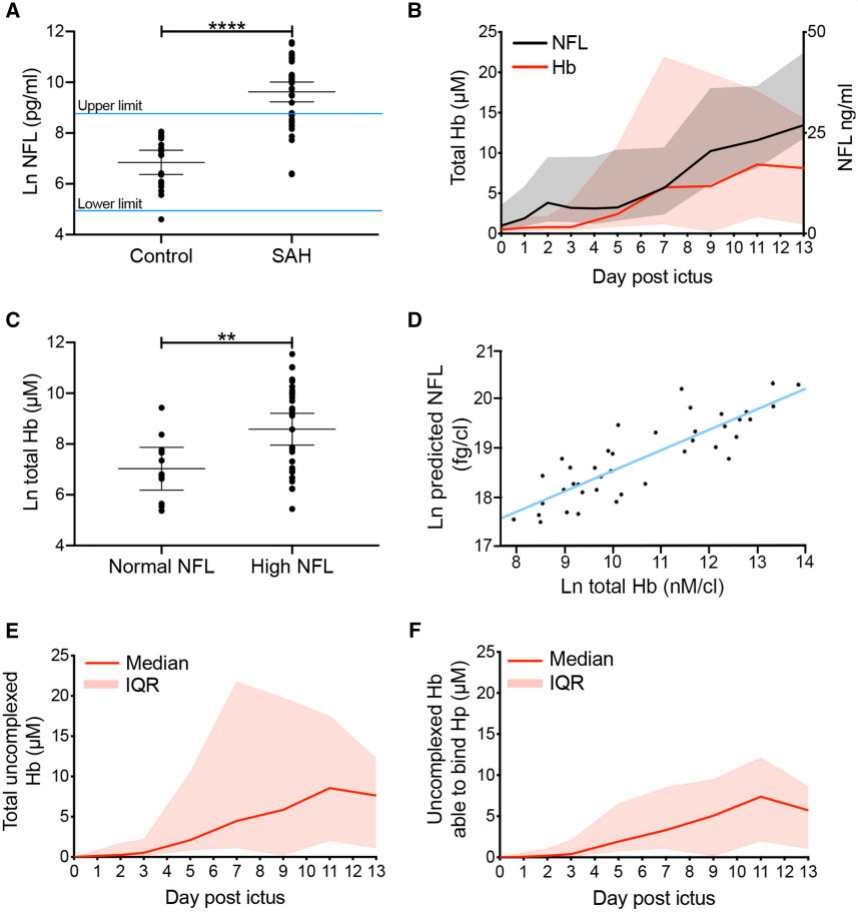
**Human study. Hb predicts NFL concentration in the CSF of SAH patients.** (**A**) Peak NFL concentration is significantly higher in the CSF of SAH patients (*n* = 42) *versus* a non-inflammatory and non-haemorrhagic control population (*n* = 19). Upper and lower limits were derived from the Ln normal distribution of the control population (mean ± 95% CI, unpaired *t*-test). (**B**) The CSF concentration of total Hb and NFL increase over a 2-week period post-ictus [*n* = 42; median ± inter-quartile range (IQR, shaded zone)]. (**C**) Peak Hb concentration preceding peak NFL concentration is significantly higher in SAH patients with high NFL (*n* = 30) *versus* SAH patients with normal NFL (*n* = 12; mean ± 95% CI, unpaired *t*-test). (**D**) Multivariable linear regression of peak NFL on preceding peak total Hb, controlling for WFNS, age and sex [*n* = 42, *B* = 0.364 (95% CI: 0.156–0.573), *P* = 0.001]. (**E**) Uncomplexed Hb (Hb not bound to Hp). (**F**) Uncomplexed Hb that can bind Hp (**E**, **F**) *n* = 42, median with IQR). (**A**, **C**) *P*-values: **P < 0.01, ****P < 0.0001.

The ideal test of the hypothesis that exposure of the brain to Hb affects outcome after SAH in humans is to measure Hb in the CSF serially and relate this to outcome. However, this poses a significant practical challenge since it is difficult to sample CSF with sufficient frequency and duration to arrive at a reliable estimate of total Hb exposure over the course of clot lysis, a process taking up to a month (Naff *et al.*, 2001). Therefore, we focussed on an initial 2-week time interval, during which we serially sampled CSF and investigated whether there was a temporal relationship between Hb and NFL, a marker of neuronal damage (Siedler *et al.*, 2014). We hypothesized that within the 2-week sampling window, peak NFL in the CSF could be predicted by the preceding peak Hb. Serial CSF samples were collected from 42 patients with Fisher grade III–IV SAH via an EVD. Control CSF was collected by lumbar puncture in patients with non-inflammatory non-haemorrhagic neurological symptoms. Demographic and clinical characteristics are shown in Table 1. NFL measured by enzyme-linked immunosorbent assay was higher in SAH CSF *versus* control CSF (Fig. 2A). This difference occurred even though there is a ventriculo-lumbar gradient in CSF NFL, with lumbar CSF concentration being higher (Jeppsson *et al.*, 2013). CSF NFL levels after SAH had a wide distribution, some within the reference range (Fig. 2A). CSF samples were analysed using UPLC for Hb species as detected by absorbance in the Soret band at 415 nm to quantify total Hb, irrespective of oxidation state (Fig. 1A and B). Total Hb increased gradually from the third-day post-ictus onwards, peaking at day 11 (Fig. 2B) and reaching a plateau between days 11 and 13. There was a small initial spike in CSF NFL level, followed by a gradual rise (Fig. 2B). The gradual rise in NFL (day 5 onwards) followed the rise in Hb (day 3 onwards; Fig. 2B). When comparing patients with high *versus* normal peak CSF NFL levels, the former had significantly higher CSF Hb levels preceding the peak CSF NFL (Fig. 2C). Peak NFL concentration in serial CSF samples was predicted by the peak Hb level preceding it, controlling for initial neurological state as determined by the World Federation of Neurological Surgeons (WFNS; Teasdale *et al.*, 1988), age and sex (Fig. 2D).

**Table 1:** Demographic and clinical characteristics of the clinical cohort

	Non-traumatic SAH patients	Control patients
Number	44	19
Age (years)^a^	59.8 ± 12.3	55.2 ± 25.9
Gender^b^		
Male	15 (34%)	5 (26%)
Female	29 (66%)	14 (79%)
Hypertension^b^		
Yes	23 (52.3%)	
No	21 (47.7%)	
Initial WFNS score^b^		
1	5 (11.4%)	
2	10 (18%)	
3	6 (13.6%)	
4	15 (29.5%)	
5	8 (13.6%)	
Fisher grade^b^		
3	2 (4.5%)	
4	42 (95.5%)	
Aneurysmal management^b^		
Coiled	32 (72.7%)	
Clipped	5 (11.4%)	
Supportive	6 (13.6%)	
Aneurysm location^b^		
Anterior circulation	32 (72.7%)	
Posterior circulation	7 (15.9%)	
Perimesencephalic	4 (9.1%)	
Unknown	1 (2.3%)	

The WFNS score describes the initial neurological condition.

a Mean and standard deviation.

b Number and %.

#### An opportunistic therapeutic window for intrathecal Hp supplementation after SAH

Hp is present at low levels in CSF (Galea *et al.*, 2012), so we set out to find the percentage of Hb complexed to Hp after SAH. Hb–Hp complexes could be differentiated from uncomplexed Hb, due to the difference in retention time on the column, and a corresponding difference in peak size (Fig. 1B). Most of the Hb was uncomplexed [median 96.3%, inter-quartile range (IQR): 83.3–99.4%, from third day onwards, Fig. 2E].

The prolonged presence of Hb in a pro-inflammatory acidotic environment is particularly conducive to denaturing modifications of Hb, rendering it less able to bind Hp (Vallelian *et al.*, 2008). Modified Hb forms have been detected in CSF after SAH (Reeder *et al.*, 2002) and their formation has the potential to interfere with the therapeutic potential of Hp. Therefore, we asked whether the uncomplexed Hb in the CSF after SAH could be bound by exogenous Hp. CSF was first run on UPLC on its own. Then exogenous Hp was added in excess of Hb-binding stoichiometry and the mixture run on UPLC to determine the proportion of uncomplexed Hb which could be bound by Hp (Fig. 1C). When exogenous Hp was added to the CSF samples, most of the uncomplexed Hb formed a complex with Hp: 90.6% (IQR: 65.8–96.5%) from the third day onwards (Fig. 2F). Similarly, 76.4% (IQR: 67.7–96.4%) of uncomplexed Hb could form a complex with Hp during the day 11–13 plateau. Therefore, most uncomplexed Hb in the CSF was able to bind Hp in the first 2 weeks after ictus. This represents a therapeutic opportunity for intrathecal Hp supplementation after SAH.

### *In vitro* study

#### Hp attenuates the toxicity of Hb towards primary mouse neurones

**Figure 3:**
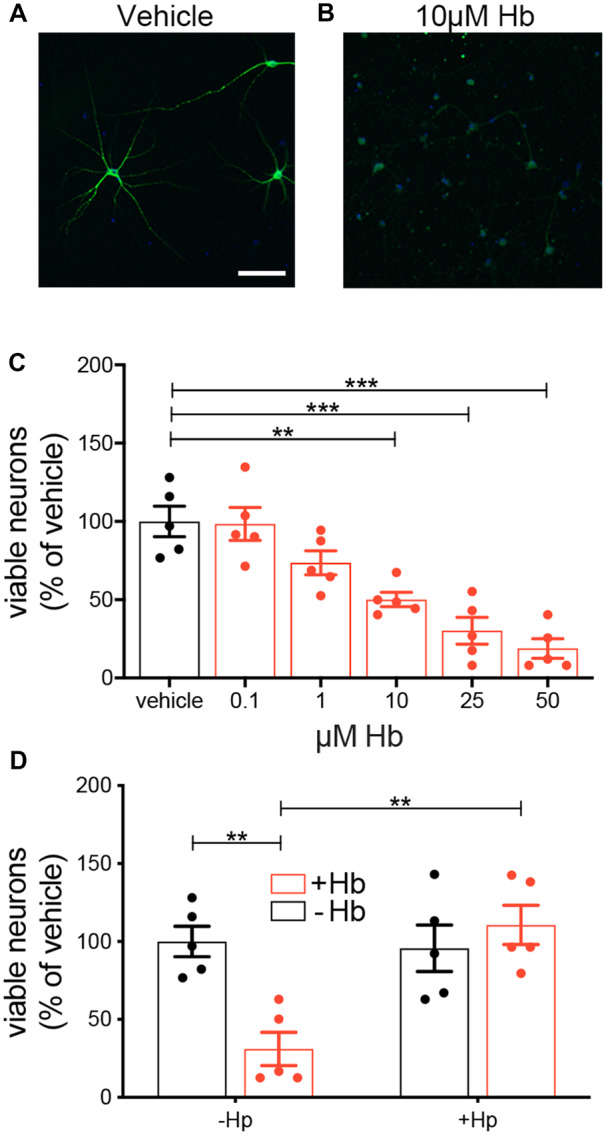
***In vitro* study. Hp reverses Hb toxicity towards mouse hippocampal neurones.** (**A**, **B**) Representative images of MAP2-positive neurones challenged for 1 week with vehicle or 10 µM mouse Hb. (**C**) Increasing concentration of Hb reduces the number of MAP2-positive neurones (*n* = 5 experiments each with duplicate wells per condition, mean ± SEM, one-way ANOVA *P* < 0.0001, *P*-values for multiple comparisons are from Dunnett’s test). (**D**) An equimolar-binding ratio of Hp fully reverses a 1-week 15 µM Hb challenge (*n* = 5 experiments each with duplicate wells per condition, mean ± SEM). Two-way ANOVA: Hb × Hp interaction *P* = 0.0032. (**A**) Scale bar = 100 μm. (**C**, **D**) *P*-values: **P < 0.01, ***P < 0.001.

Next, we wanted to confirm that Hp can protect against Hb toxicity. The toxicity of Hb to cortical neurones in mixed glial cultures from foetal mice has been observed previously (Regan and Panter, 1993). We used a protocol that allows culturing of primary hippocampal neurones from mouse pup brain at low density (Fath *et al.*, 2009; Garland *et al.*, 2012; Fig. 3A). Microtubule-associated protein 2 (MAP2) positive neurones with 4ʹ,6-diamidino-2-phenylindole, dihydrochloride (DAPI)-positive non-apoptotic nuclei (hence viable neurones) were counted following a 1-week challenge with increasing concentrations of mouse Hb. The number of viable neurones was found to be reduced after exposure to Hb concentrations above 1 μM (Fig. 3A–C). Concomitant addition of an equimolar amount of Hp completely neutralized this Hb toxicity (Fig. 3D).

### *In vivo* animal model

#### Prolonged intrathecal exposure to Hb causes behavioural deficits, which are attenuated by Hp

**Figure 4:**
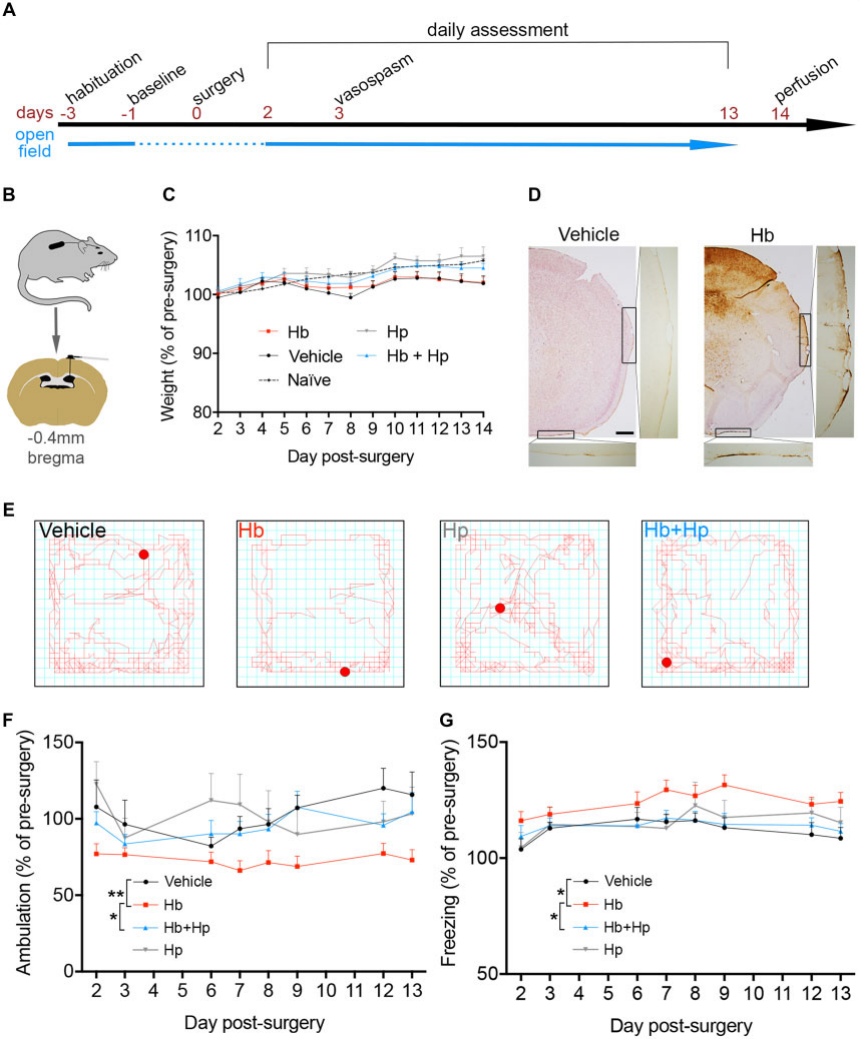
***In vivo* animal model: behaviour. Hp reverses behavioural deficits resulting from intra-cerebroventricular infusion of Hb.** (**A**) Experimental timeline. Mice were habituated to the open-field and weighing apparatus for 3 days prior to surgery. Data from the day before surgery were taken as baseline. Post-surgery weight and open-field behaviour were assessed from day 2 until day 13. (**B**) Ventricular cannula and osmotic minipump placement. (**C**) Daily assessment of animal weights following surgery over the 2-week experiment (saline: *n* = 10, Hb: *n* = 13, Hb + Hp: *n* = 13, Hp: *n* = 9). (**D**) Representative images of Perls’ staining, showing iron deposition. (**E**) Representative traces following a 5-min open-field assessment. (**F**) Linear-mixed modelling analysis of time-series data for ambulation performance in the open field (saline: *n* = 11, Hb: *n* = 16, Hb + Hp: *n* = 15, Hp: *n* = 9). Hb × Hp interaction *P* = 0.049. (**G**) Linear-mixed modelling analysis of time-series data for freezing, i.e. period spent immobile in the periphery of the open-field arena in the first minute (saline: *n* = 11, Hb: *n* = 16, Hb + Hp: *n* = 15, Hp: *n* = 9). Hb × Hp interaction *P* = 0.065. (**C**, **F**, **G**) Mean ± SEM. (**F**, **G**) *P*-values: *P < 0.05, ***P < 0.001. Scale bar = 500 μm.

A therapeutic potential for Hp has been strongly suggested by experiments in an animal model of focal intraparenchymal cerebral haemorrhage, in which Hp overexpression reversed some of the acute neurological deficits after a large (12 µl) intrastriatal injection of whole blood (Zhao *et al.*, 2009). This study provided valuable information, yet the mechanical and inflammatory consequences of a large intraparenchymal injection, and the associated plasma proteins, cell membranes, and white cell constituents, meant that it could not be definitely concluded that Hp targeted the Hb component. Moreover, in the same study Hp expression was manipulated indirectly *in vivo* (Zhao *et al.*, 2009), making it hard to be absolutely certain that Hp was the neuroprotective agent. In order to model the chronic Hb exposure aspect of SAH in isolation, as opposed to the whole SAH condition, adult male C57BL6 mice underwent insertion of an intraventricular cannula attached to a subcutaneously implanted osmotic minipump; this protocol delivered Hb slowly over 2 weeks (Fig. 4A and B). Male mice were used since (i) sex did not affect Hb neurotoxicity in the human studies [Fig. 2D, *n* = 42, *B* = 0.044 (95% CI:−0.742 to 0.822), *P *=* *0.918] and (ii) variability in planned behavioural measures is greater in females, due to the oestrous cycle (Gray and Cooney, 1982). The concentration of Hb was selected to achieve a clinically relevant concentration of 10 μM, based on the median total Hb observed in CSF from SAH patients. *In vitro* incubation of Hb or Hb + Hp complexes at 37°C for 2 weeks showed that Hb did not degrade to release iron, as measured by a free iron assay (Supplementary Fig. 1). The general health of animals in this model was assessed, including daily measurements of weight (Fig. 4C). Perls’ staining of brain sections showed iron deposition within the parenchyma and over the convexities (Fig. 4D), the latter confirming circulation of Hb out of the ventricles, into the subarachnoid space, and over the outer surface of the brain.

An automated open-field arena was used to assess mouse ambulatory activity; animals were tested between days 2 and 13 post-surgery and compared relative to their pre-surgery baseline (Fig. 4E–G). Linear-mixed modelling of this time-series data revealed a significant reduction in ambulation in animals challenged with 10 μM Hb compared with the vehicle control (Fig. 4F, Supplementary Fig. 2). However, concomitant infusion of Hp with Hb recovered this behavioural deficit (Fig. 4F, Supplementary Fig. 2).

Longer periods of immobility, so-called freezing, in the periphery of the open-field have been described previously as a symptom of anxiety (Simon *et al.*, 1994). Therefore, zonal analysis of the open-field data was performed to measure the time mice spent freezing in the periphery over the first minute (Fig. 4G). A significant increase in freezing was observed in animals challenged with 10 μM Hb compared with vehicle. This recovered following concomitant treatment with Hp (Fig. 4G).

#### Prolonged intrathecal exposure to Hb causes a glial reaction and synaptic loss, which are attenuated by Hp

**Figure 5:**
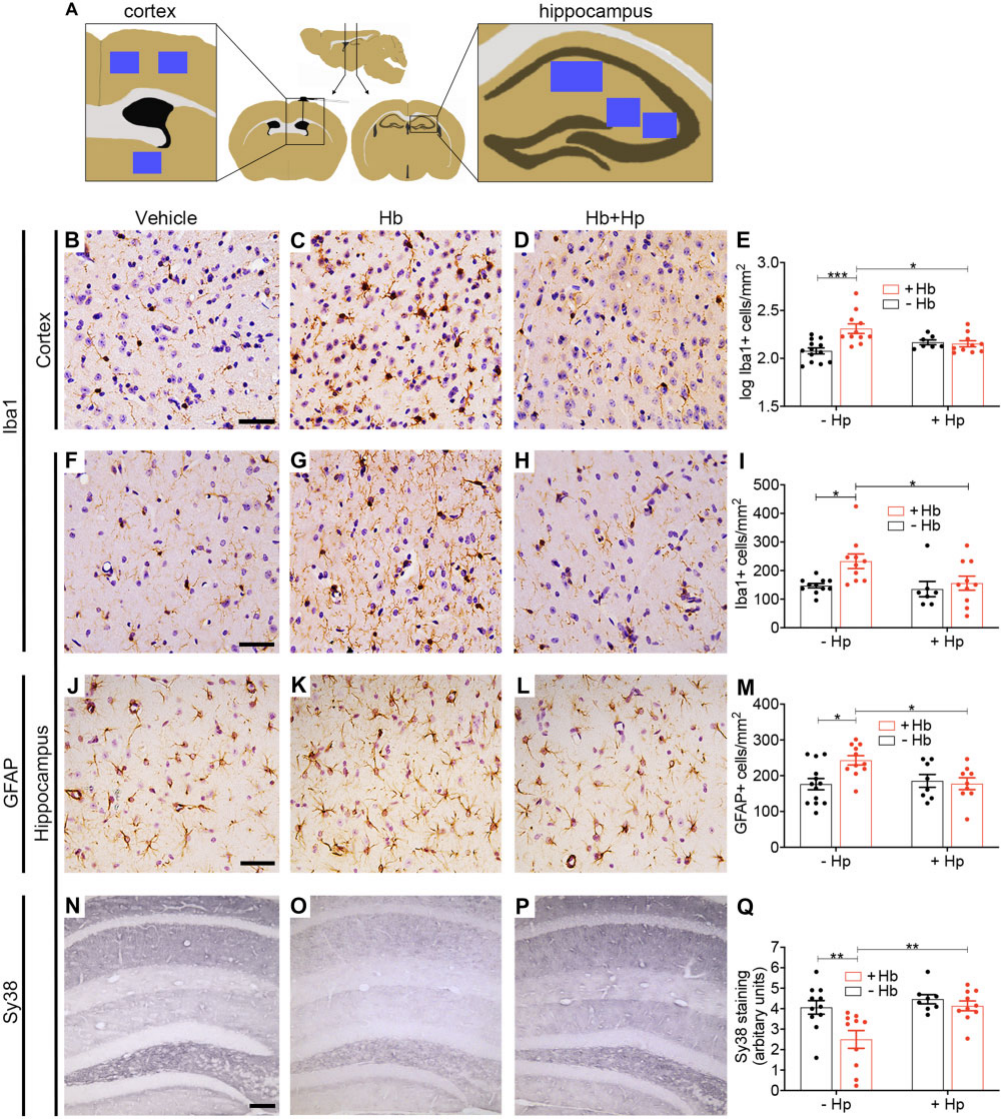
***In vivo* animal model: histology. Hp reverses changes in immunohistochemical markers of inflammation, injury and synapse loss following prolonged intrathecal Hb exposure.** (**A**) Sampling sites (blue squares). (**B**–**E**) Log(10) transformed counts of Iba1-positive cells in the cortex show an increase following Hb infusion, which was reduced to control levels with Hp treatment (*n* = 7–13 per condition). Two-way ANOVA: Hb × Hp interaction *P* = 0.003. (**F**–**I**) Iba1-positive cells in the hippocampal molecular region (*n* = 7–11 per condition). Two-way ANOVA: main effect for Hb *P* = 0.04. (**J**–**M**) GFAP-positive cells in the hippocampal molecular region show an increase following Hb infusion, which is reduced to control levels by Hp treatment (*n* = 8–13 per condition). Two-way ANOVA: Hb × Hp interaction *P* = 0.026. (**N**–**Q**) Sy38 (synaptophysin) staining across the hippocampal layers shows a reduction after Hb, which is reversed by Hp treatment (*n* = 8–11 per condition). Two-way ANOVA: Hb × Hp interaction *P* = 0.07. (**B**, **F**, **J**) Scale bar = 50 µm. (**N**) Scale bar = 100 µm. (**E**, **I**, **M**, **Q**) Mean ± SEM, *P-*values: *P < 0.05, **P < 0.01, ***P < 0.001.

Following behavioural assessment, brain tissue was collected for immunohistochemistry. Two main brain parenchymal regions were selected for quantification (Fig. 5A), close to and distal from the infusion site (cortical and hippocampal regions respectively). The microglial/macrophage response to intrathecal Hb was assessed using immunohistochemistry for Iba1, a cytoplasmic protein constitutively expressed by microglia and upregulated during inflammation (Ohsawa *et al.*, 2004). In the cortical region, Iba1-positive cells were more numerous after Hb challenge compared with vehicle challenge (Fig. 5B, C and E). Concomitant infusion of an equimolar-binding amount of Hp with Hb reduced the number of Iba1-positive cells (Fig. 5D and E). A similar pattern was observed caudally, in the glia-rich hippocampal molecular region (Fig. 5F–I) and throughout the hippocampus (Supplementary Fig. 3A).

The astrocytic response to injury from intrathecal Hb was assessed using GFAP. Compared with vehicle, animals challenged with Hb had more GFAP-positive cells in the hippocampal molecular region; this was reversed by concomitant treatment with Hp (Fig. 5J–L). A similar trend was present throughout the hippocampus (Supplementary Fig. 3B) and cortex (Supplementary Fig. 3C).

We investigated hippocampal synaptic integrity using Sy38, an antibody against the pre-synaptic protein synaptophysin (Fig. 5N–Q). Synaptic loss was observed in Hb animals, relative to vehicle (Fig. 5N, O and Q). Concomitant infusion of Hp with Hb increased Sy38 staining back to control levels (Fig. 5P and Q). A trend towards an interaction between Hb and Hp was found for this marker (*P* = 0.07); significant main effects were observed (Supplementary Table 1). A similar picture was observed when tissue was analysed for postsynaptic density protein 95 (PSD-95) by western blotting (Supplementary Fig. 4).

#### CD163 expression

**Figure 6:**
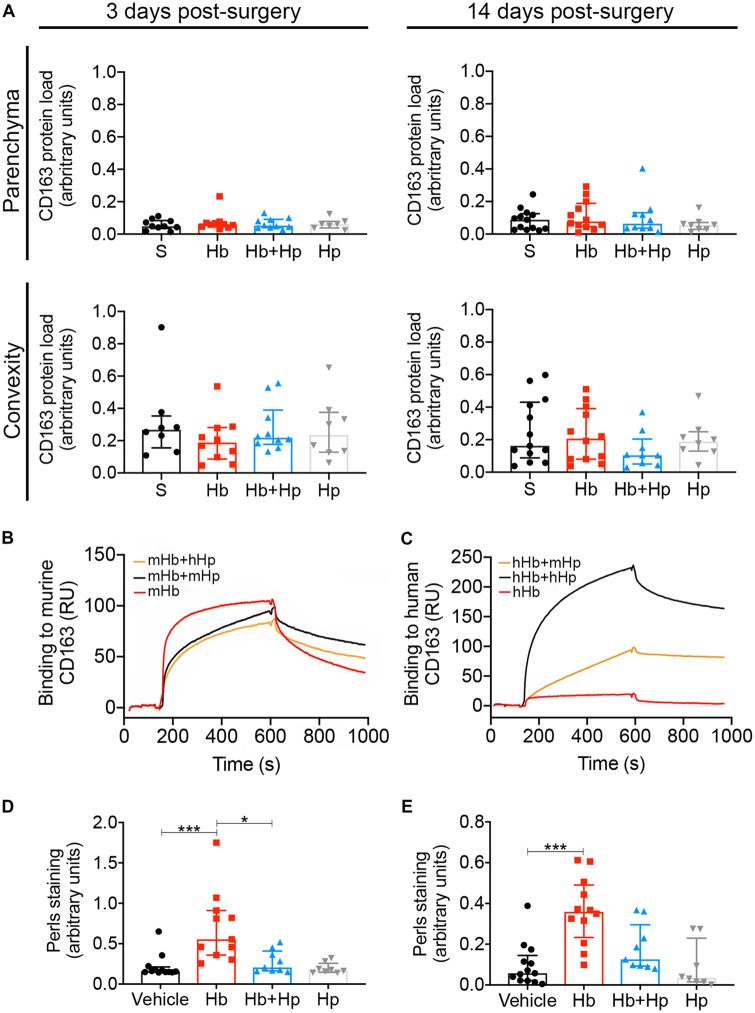
***In vivo* animal model: CD163 and iron. *Top panel*—Immunohistochemical analysis of CD163 expression.** (**A**) At 6 and 14 days post-surgery, both parenchymal and convexity staining for CD163 was unchanged between experimental groups (*n* = 8–13, median ± IQR). *Middle panel*—Assessment of mixed-species Hp to Hb binding. (**B**, **C**) Surface plasmon resonance analysis shows human Hp (hHp) does not augment binding of mouse Hb (mHb) to mouse CD163 (**B**), but hHp does increase binding of human Hb (hHb) to human CD163 (**C**). *Bottom panel*—Hp reverses Perls’ staining following intrathecal infusion of Hb. (**D**) Perls’ staining in the ipsilateral parenchyma at 14 days is significantly increased following Hb infusion, which is fully reversed by Hp (*n* = 8–12, Kruskal–Wallis *P* = 0.0002, median ± IQR). (**E**) Perls’ staining on the brain surface and outer layers of the cortex on the ipsilateral side is significantly increased following Hb infusion (*n* = 8–13, Kruskal–Wallis *P* = 0.0003, median ± IQR), which is decreased by Hp. (**D**, **E**) *P-*values: *P < 0.05, ***P < 0.001.

CD163 is the Hb receptor (Kristiansen *et al.*, 2001) and its expression is increased after uptake of Hb (Boyle *et al.*, 2009; Liu *et al.*, 2017). CD163 expression in the brain is very limited (Galea *et al.*, 2012), so it was important to determine whether the Hp therapeutic effect relied on CD163. In order to determine whether the therapeutic effect of Hp was mediated via upregulation of CD163 expression, we used immunohistochemistry. CD163 expression was seen in the meninges and perivascular spaces, as is usual in normal brain (Galea *et al.*, 2008). We did not observe robust changes in CD163 expression after infusion of Hb or Hb–Hp complexes, at either 3 or 14 days in the parenchyma or convexity (Fig. 6A). Also, surface plasmon resonance demonstrated that human Hp does not increase binding of mouse Hb to mouse CD163 (Fig. 6B), in contrast to the human scavenging system (Fig. 6C). In summary, the Hp therapeutic effect did not rely on CD163.

#### Hp prevents iron deposition

When Hb is oxidized and subsequently degrades, free chelatable iron is released (Asleh *et al.*, 2005; Reeder, 2010). Deposition of this iron in tissue can be visualized using Perls’ stain, which we have observed to be increased in post-mortem brain tissue from SAH patients (Garland *et al.*, 2016). Similarly, iron deposition was observed in the parenchyma (Fig. 6D) and on the brain surface and outer layers of the cortex (Fig. 6E) in Hb-challenged animals relative to vehicle. In both locations, Hp treatment reduced iron deposition (Fig. 6D and E). Positive correlations were observed between the Perls’ staining and: microglia (Iba1, Supplementary Fig. 5A, B, G and H), astrocytes (GFAP, Supplementary Fig. 5C and I), and freezing (Supplementary Fig. 5F and L). Negative correlations were observed between the Perls’ staining and Sy38 staining in the hippocampus (Supplementary Fig. 5D and J) and ambulation (Supplementary Fig. 5E and K).

#### Hp prevents diffusion of Hb into the parenchyma and attenuates small-vessel, but not large-vessel, vasospasm

**Figure 7:**
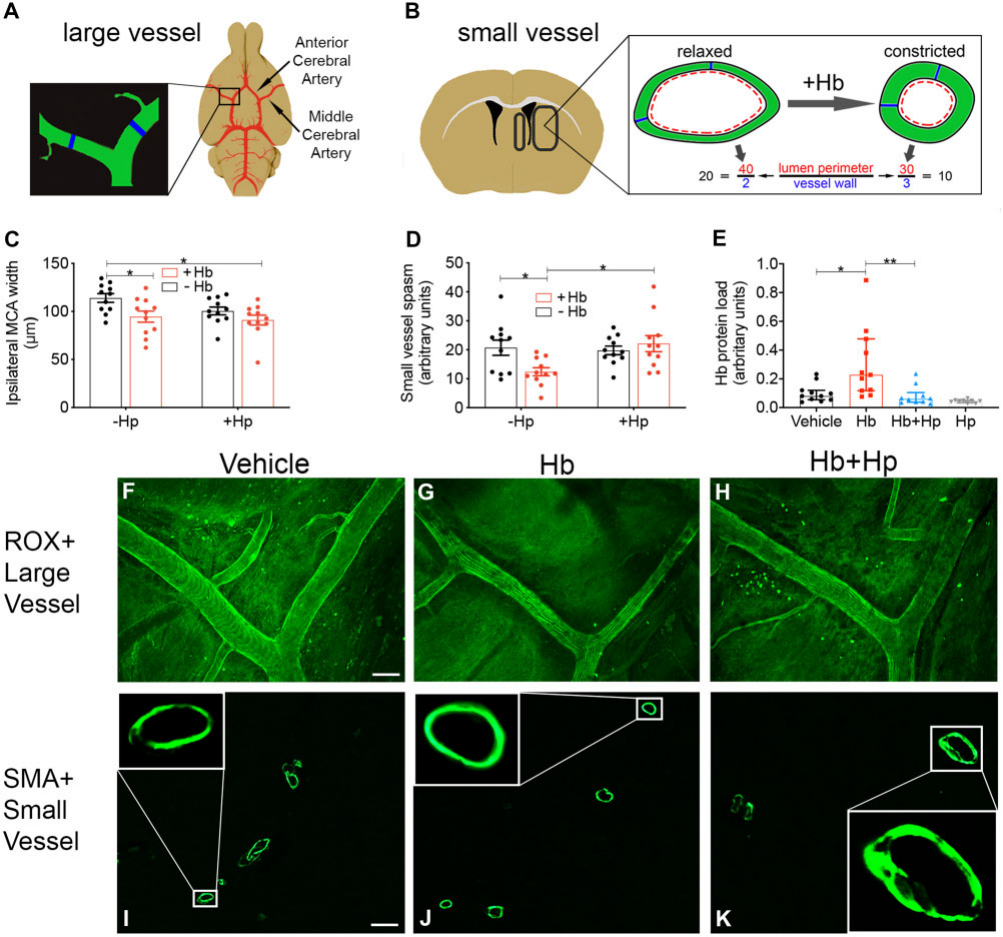
***In vivo* animal model: vasospasm. *Large vessel*—Intrathecal infusion of Hb for 3 days results in vasospasm of the MCA.** (**A**) Illustration of Circle of Willis and sampling area. (**C**) Hb decreases ipsilateral MCA diameter, and this is unaffected by Hp. Two-way ANOVA: main effect Hb = 0.0058 (*n* = 11, mean ± SEM). (**F**, **G**, **H**) Representative images of MCA *in situ*. Scale bar = 100 μm. (**C**) *P*-value: *P < 0.05. *Small vessel*—Hp reverses small-vessel vasospasm resulting from intrathecal infusion of Hb. (**B**) Illustration of parenchymal sampling areas and method to determine vasoconstriction. (**D**) Hp significantly reverses vasospasm in ipsilateral SMA+ vessels caused by a 3-day intrathecal Hb infusion. Two-way ANOVA: Hb × Hp interaction *P* = 0.0182 (*n* = 11, mean ± SEM). (**E**) Immunohistochemical staining for Hb reveals a significant increase in the ipsilateral parenchyma after a 3-day Hb infusion, which is fully reversed by Hp (*n* = 10–11, Kruskal–Wallis *P* = 0.0001, median ± IQR). (**I**–**K**) Representative confocal images of SMA+ vessels in the ipsilateral parenchyma. Inset images are confocal slices at higher magnification (×20). Scale bar = 50 μm. (**D**, **E**) *P-*values: *P < 0.05, **P < 0.01.

Angiographic large-vessel vasospasm and long-term outcome are dissociated (Diringer, 2013), interventions which attenuate large-vessel vasospasm have proved unsuccessful (Cho *et al.*, 2019) and interventions which improve long-term outcome do not affect large-vessel vasospasm (Pickard *et al.*, 1989). However, dysregulation of microcirculation following SAH is an emerging area of study (Terpolilli *et al.*, 2015). As Hb is a known mediator of vasospasm, we hypothesized that the Hb challenge in our model would promote large and small-vessel vasospasm, and this would be attenuated by Hp. Following a 3-day infusion of Hb, anaesthetized mice were transcardially perfused with 5-(6)-carboxy-X-rhodamine, succinimidyl ester for *in situ* imaging of the Circle of Willis (Fig. 7A, C and F–H). A reduction in MCA diameter was observed with Hb *versus* vehicle and this was not reversed by Hp (Fig. 7C). A similar picture (Hb-induced vasospasm, not reversed by Hp) was seen with other cerebral arteries (Supplementary Fig. 6). To study parenchymal microcirculatory changes, the same brains used for large-vessel imaging were then prepared for immunohistochemistry and stained for SMA (Fig. 7B, D, I and K). The ratio of vessel lumen diameter to wall thickness was used as a metric of vasospasm (Sabri and Macdonald, 2012; Fig. 7B). Hb caused vasospasm which was reversed by Hp. Two-way ANOVA revealed a significant interaction between Hb and Hp (Fig. 7D). *Posthoc* analysis revealed significant vasospasm of SMA+ vessels with Hb compared with vehicle, and a significant recovery with Hp (Fig. 7D and I–K).

Hb may cause vasospasm via decreasing nitric oxide (Kanamaru *et al.*, 1987; Lin *et al.*, 2001), yet Hb–Hp complexes bind nitric oxide with similar affinity to Hb alone (Azarov *et al.*, 2008). After Hb infusion, vasospasm occurred in both large and parenchymal vessels but Hp only reversed vasospasm in parenchymal vessels. Hence, it was possible that Hp was influencing the distribution of Hb in the brain. Complexation by Hp, especially the polymeric form, results in sequestration of Hb in moieties with a molecular weight of up to 1760 kDa, which would severely restrict entry of Hb into the brain tissue. The movement of solutes into the brain is size-selective; single-domain antibody (17 kDa) penetrates brain tissue better than whole immunoglobulin G (150 kDa; Pizzo *et al.*, 2018), and movement of ovalbumin-immunoglobulin G immune complexes (500–2000 kDa; Mosconi *et al.*, 2010) within brain tissue is significantly limited (Carare *et al.*, 2013). To test the hypothesis that Hp was sequestering Hb outside brain tissue, we assessed the Hb content of the brain parenchyma using immunohistochemistry. At 3 days post-surgery, the same time point as the observed microcirculatory vasospasm, Hb staining in the ipsilateral parenchyma was significantly increased after Hb infusion, compared with vehicle. Hp reversed this parenchymal increase in Hb content (Fig. 7E).

## Discussion

SAH results in the prolonged presence of Hb in the subarachnoid space. In humans after SAH, we followed the course of Hb and a neuronal damage marker over an initial discrete observation window. NFL level in the CSF rose gradually, indicating a prolonged stimulus, as opposed to an acute rise immediately after ictus. CSF Hb concentration was low in the first 2 days, but subsequently increased to a plateau after 10 days, likely due to the cumulative effect of red blood cell lysis. CSF NFL level followed this rise in Hb with a lag of 2 days. CSF NFL and preceding Hb peaks were significantly related. NFL was used as a marker for Hb-induced tissue damage, hence the mechanisms underlying NFL release after exposure to Hb were not investigated here; these may include direct neuronal damage or secondary consequences such as inflammation.

While it is well-known that SAH results in the prolonged presence of Hb in the subarachnoid space, the pathophysiological consequences of prolonged *in vivo* exposure to Hb have not been investigated in isolation. We confirmed previous observations that Hb is toxic to cultured neurones at clinically relevant concentrations (Regan and Panter, 1993; Zhao *et al.*, 2009). In order to establish a clinically relevant model of prolonged Hb exposure, one needs to consider that SAH survivors experience cognitive, behavioural and emotional deficits (Al-Khindi *et al.*, 2010). Modelling these functional outcomes in pre-clinical research is important (Turan *et al.*, 2017). In both the endovascular perforation and blood injection models of SAH, the open-field paradigm has shown significant changes in ambulation and anxiety (Boyko *et al.*, 2013; Fanizzi *et al.*, 2017). However, it was not known whether prolonged exposure to Hb alone—without the mechanical and ischaemic tissue injury, and potential effects of other blood constituents—could independently contribute to, or suffice for, the occurrence of these behavioural deficits. In this study, we have shown that a 2-week intrathecal exposure to Hb does in fact lead to behavioural deficits similar to those seen in clinic, evidenced by reduced ambulation and increased anxiety. A loss of synaptic integrity, as suggested by immunohistochemistry for synaptophysin, accompanied these behavioural deficits. In response to Hb exposure, accumulation of macrophages/microglia was observed in this model, as happens after human and experimental SAH (Schneider *et al.*, 2015). The increase in GFAP-positive cells seen here parallels the high levels of astrocyte-derived serum GFAP and S100β after human SAH, especially observed in patients with poor outcome (Vos *et al.*, 2006; Lai and Du, 2016). Vasospasm was observed in both large cerebral arteries and arterioles in the parenchyma, as seen in human SAH (Terpolilli *et al.*, 2015; Macdonald and Schweizer, 2017). In summary, we observed behavioural, vascular, cellular and molecular changes strikingly similar to those after SAH, by purely modelling one single aspect of SAH: the prolonged intrathecal exposure to uncomplexed Hb.

This study provides robust and conclusive evidence that uncomplexed Hb is a promising therapeutic target in haemorrhagic brain conditions. Hp can reverse Hb toxicity (Zhao *et al.*, 2009), yet Hp concentration is very low in the brain (Galea *et al.*, 2012). We found that most Hb present in human CSF after SAH was uncomplexed. Most of this uncomplexed Hb retained the capacity to bind exogenously added Hp throughout the first 2 weeks. Therefore, there is a wide therapeutic window for treatments based on administering or upregulating Hp in the CNS after SAH. In an *in vivo* model, Hp supplementation was well-tolerated and reversed the neurological deficit occurring in response to Hb. In addition, Hp reduced the microglial and astrocytic reaction to Hb and synaptic loss.

We did not observe robust changes in CD163 expression accompanying the therapeutic effect of Hp in this model. In mouse, Hp is not required for binding of Hb to CD163 (Etzerodt *et al.*, 2013), unlike in humans, where the Hb–Hp complex has a 10-fold higher affinity to the scavenging receptor CD163 relative to Hb alone (Kristiansen *et al.*, 2001). Human Hp employed in these experiments did not increase the affinity of mouse Hb to mouse CD163. Taken together these findings suggest that the therapeutic effect of Hp was not mediated by increased Hb uptake. Instead, Hp stabilized Hb, preventing its degradation and subsequent release of iron. Iron deposition correlated with behavioural deficits, immunohistochemical markers of an astrocytic and microglial reaction and synaptic loss. Hence, clinically relevant Hp protection can be achieved solely by stabilizing Hb in solution, independent of clearance by CD163. This is particularly encouraging since there is evidence that CD163 binding sites in the human brain are much reduced when compared with the periphery, and fully saturated after SAH (Galea *et al.*, 2012), such that Hb–Hp complexes queue for clearance. Endogenous clearance of CSF into the bloodstream and extracerebral lymphatics occurs via arachnoid granulations, cribriform plate and cranial/spinal nerve roots (Kida *et al.*, 1993; Aspelund *et al.*, 2015). The results in this study confirm that there is therapeutic potential for Hp supplementation despite saturation of CD163 binding sites after SAH.

Hp treatment reduced parenchymal Hb staining, suggesting that another mechanism of action of Hp is to reduce the bioavailability of Hb by sequestering it in large complexes in the subarachnoid space and therefore limiting its diffusion into brain tissue. Hp treatment did not attenuate large-vessel vasospasm, yet improved clinical outcome. Instead, Hp attenuated small-vessel vasospasm. Hence, we experimentally confirm that clinical outcome can be improved independently of large-vessel vasospasm, in keeping with the clinically observed dissociation between clinical outcome and angiographic vasospasm.

Haem released by Hb degradation is bound by haemopexin, and the complex is cleared by CD91 receptor-mediated endocytosis (Hvidberg *et al.*, 2005), providing a natural defence mechanism against haem. Both haemopexin and CD91 are expressed by neurones and glia (Moestrup *et al.*, 1992; Morris *et al.*, 1993). Haemopexin, in contrast to Hp, has a much higher concentration in CSF than anticipated from its plasma concentration (Garland *et al.*, 2016). This suggests that haemopexin may mediate protection from haem. However a higher CSF haemopexin level was associated with a worse outcome after SAH (Garland *et al.*, 2016), and it has been shown *in vitro* that haemopexin increases the neurotoxicity of Hb when Hp is absent (Chen-Roetling *et al.*, 2018). It may be possible to improve the therapeutic efficacy of Hp by co-administration with haemopexin.

Future work is needed to determine whether Hp can produce a clinically relevant improvement after SAH in animal models such as prechiasmatic blood injection (Sabri *et al.*, 2009), cisternal blood injection or endovascular perforation models (Marbacher *et al.*, 2010). Ideally, this modelling is performed in a CD163-humanized mouse model, in view of the marked differences in CD163 biology between mouse and man (Etzerodt *et al.*, 2010; Etzerodt *et al.*, 2013). Another suitable animal model would be the pig, in which Hp increases the affinity of Hb to CD163, as in the human system (personal communication, J.H. Graversen and S.K. Moestrup). A Phase I trial is needed to ensure that Hp administration is safe in humans. It has been suggested that Hp increases the vulnerability of murine CD163-expressing neurones to Hb *in vitro* (Chen-Roetling and Regan, 2016), but this was not observed in our *in vitro* experiments. In a mouse model of intracerebral haemorrhage, CD163 appeared to be detrimental in the first 4 days after the bleed but then conferred protection after 4 days (Leclerc *et al.*, 2018). This inflection may represent a change in the ratio of CD163 expression by neurones *versus* glia/macrophages (Jing *et al.*, 2018), since myelomonocytic ingress into the CNS is delayed by a few days after an inflammatory stimulus (Andersson *et al.*, 1992).

There are several possible Hp-based therapeutic approaches. Direct intrathecal delivery of Hp after high-grade SAH would be feasible by the time Hb starts rising in the CSF, since by then patients are usually in a tertiary neurological centre, and likely to have an EVD *in situ*. Hence, Hp and Hb would also be present at the same time in the human situation, as modelled with the osmotic minipump here. The challenge in the human situation relates to ensuring that Hp and Hb are present in the same place, i.e. that intrathecal Hp dosing is such that it reaches the far corners of the subarachnoid space, to ensure that Hb is bound by Hp as soon as it is released. The mouse is not the right species to model the intricacies of Hp dosing in humans in view of different subarachnoid space anatomy, CSF turnover rate and drainage pathways. High purity clinical-grade Hp is available, though all preparations available at the moment are a mixture of dimeric and polymeric Hp. In these experiments, we used a similar preparation, though it was enriched in dimeric Hp. Future work needs to address the issue of which Hp isoform is most efficacious, especially in view of reported differences in clinical outcome between SAH patients with different *HP* genotypes (Gaastra *et al.*, 2019). It may be preferable to determine an individual’s Hp genotype and administer the same Hp type, to prevent unwanted immunological rejection in a brain compartment (the subarachnoid space) which is not immunologically privileged (Galea *et al.*, 2007). Spectrophotometric Hb assays which can be easily adapted to achieve a quantitative measure of Hb concentration (Duiser *et al.*, 2001) are clinically available in most hospitals, and using serial CSF Hb assays, it may be possible to alter the Hp dose on a patient-to-patient and day-to-day basis, to deliver the right amount of Hp at the right time. Our observations that Hp–Hb binding is sufficient for therapeutic efficacy, independent of CD163 or brain penetration, suggest that an alternating intrathecal Hp infusion and CSF drainage protocol may be appropriate. Other possible therapeutic avenues are drugs which upregulate Hp production such as nuclear factor-erythroid 2 (NF-E2)-related factor 2 (nrf2) inducers (Zolnourian *et al.*, 2019).

In summary, this study provides several novel lines of insight into SAH pathophysiology. In CSF, the time course of the tissue damage marker NFL followed that of Hb. We confirm experimentally that Hb on its own is sufficient to produce an array of behavioural, vascular, cellular and molecular changes strikingly similar to those after human SAH. Robust evidence is presented here that Hp protects against Hb toxicity *in vivo*, mediated via stabilization of Hb and reduction in iron deposition, and by preventing the entry of Hb into brain tissue. We confirm experimentally the clinically observed dissociation between angiographic vasospasm and clinical outcome, in favour of an effective treatment. As treatment options are limited for SAH, further work is needed to establish Hp as a therapy. The observations regarding haptoglobin efficacy and mechanism of action may apply to other haemorrhagic conditions.

## Supplementary material

Supplementary material is available at *Brain Communications* online.

## Supplementary Material

fcz053_Supplementary_Data

## Abbreviations

CI: confidence interval
EVD: external ventricular drain
GFAP: glial fibrillary acidic protein
Hb: haemoglobin
Hp: haptoglobin
Iba1: ionized calcium-binding adapter molecule 1
IQR: inter-quartile range
MCA: middle cerebral artery
NFL: neurofilament light chain
PBS: physiological-buffered saline
SAH: subarachnoid haemorrhage
SEM: standard error of the mean
SMA: smooth-muscle actin
UPLC: ultra-performance liquid chromatography
WFNS: World Federation of Neurological Surgeons
