# Supplementary material for: Haemoglobin causes neuronal damage in vivo which is preventable by haptoglobin
Source: Brain Commun. 2020 Jan 3;2(1):fcz053. doi: 10.1093/braincomms/fcz053 (PMC7188517; doi:10.1093/braincomms/fcz053)

Supplementary Material for

Haemoglobin causes neuronal damage *in vivo* which is preventable by haptoglobin

Patrick Garland[1], Matthew J Morton[1,*], William Haskins[1,*], Ardalan Zolnourian[2], Andrew Durnford[2], Ben Gaastra[2], Jamie Toombs[3,4], Amanda J Heslegrave[3,4], John More[5], Azubuike I Okemefuna[5], Jessica L Teeling[6], Jonas H Graversen[7], Henrik Zetterberg[3,4,8,9], Soren K Moestrup[7,10,11,#], Diederik O Bulters[2,#], Ian Galea[1,2,#,ψ]

* these authors contributed equally
[#] joint senior authors
[ψ] corresponding author: I.Galea@soton.ac.uk

[1] Clinical Neurosciences, Clinical & Experimental Sciences, Faculty of Medicine, University of Southampton, Southampton, United Kingdom
[2] Wessex Neurological Centre, University Hospital Southampton NHS Foundation Trust, Southampton, United Kingdom
[3] UK Dementia Research Institute, University College London, UK
[4] Department of Neurodegenerative disease, Institute of Neurology, London, UK
[5] R&D, Bio Products Laboratory Limited, Elstree, Hertfordshire, United Kingdom
[6] Biological Sciences, Faculty of Environmental and Life Sciences, University of Southampton, Southampton, United Kingdom
[7] Department of Molecular Medicine, University of Southern Denmark, 5000 Odense, Denmark
[8] Department of Psychiatry and Neurochemistry, Institute of Neuroscience and Physiology, The Sahlgrenska Academy at the University of Gothenburg, Mölndal, Sweden
[9] Clinical Neurochemistry Laboratory, Sahlgrenska University Hospital, Mölndal, Sweden
[10] Department of Clinical Biochemistry and Pharmacology, Odense University Hospital, 5000 Odense, Denmark
[11] Department of Biomedicine, Aarhus University, Aarhus, Denmark

This PDF file includes, in this order:

Supplementary Materials and Methods
Supplementary References
Supplementary Table 1
Supplementary Figures 1 to 10

Supplementary Materials and Methods

Analysis of Hb in CSF from SAH patients

Human studies have been performed in accordance with the ethical standards as laid down in the 1964 Declaration of Helsinki. 44 Fisher grade III-IV SAH patients, requiring an external ventricular drain (EVD) as part of their clinical management, were recruited (National Research Ethics Committee approval number 12/SC/0666). CSF was obtained from the EVD on alternate days until removal of the EVD on day 13 (or earlier if clinically indicated). CSF was sampled on alternate days. Four patients died during the study and one of these patients had one CSF sample taken only, so this patient was not included in the analysis. Another patient had the EVD removed very early such that only one CSF sample was taken, and therefore this patient was not included either. Hence only 42 patients contributed to the study. CSF was drawn from a three way tap connecting the ventricular catheter (approximately 30cm long) to the tubing leading to an external CSF drainage and monitoring system (Becker®, Medtronic). For sampling, the tap was opened to the ventricular catheter, and closed to the drainage system. The first 3ml of CSF (representing dead space) was discarded to ensure fresh CSF was obtained. CSF was centrifuged at 1500 rcf for 10 minutes at 21°C and frozen within one hour of sampling. UPLC was used to separate CSF components in a tris-saline mobile phase. Eluate absorbance was measured at 415 nm to identify haem-containing species. CSF from individuals undergoing lumbar puncture for non-haemorrhagic neurological indications (National Research Ethics Committee reference no: 11/SC/0204) and a standard Hb solution (Sigma-Aldrich, Gillingham, Dorset, UK) confirmed the specificity of absorbance at 415nm to haem-containing species. The identity of the peaks on the UPLC chromatogram was confirmed by running the following reagents in combination with Hb/hemin: haemopexin (Sigma-Aldrich, Gillingham, Dorset, UK), Hp (Bio Products Laboratory Ltd. (BPL), Elstree, UK) and sera from Hp1-1, 2-1 and 2-2 individuals whose Hp phenotype was determined by western blotting (Galea *et al.*, 2012). To quantify Hb, a standard curve (nine data points from 0 to 1 g/l) was prepared from lyophilized human Hb (Sigma-Aldrich, Gillingham, Dorset, UK) reconstituted to 1g/L in diluent (9 g/L NaCl, 10 mM EDTA), and absorbance measured at 415 nm after UPLC. The concentration of the standard Hb solution was verified independently by spectrophotometric quantification at 570 nm using Hemocue™ (Hemocue, Sweden). Hb was measured in CSF before and after saturating with Hp (BPL, Elstree, UK) to measure Hp-bindable and Hp-unbindable uncomplexed Hb (Fig. 1). 50μl of each sample was loaded onto

the UPLC column (Acquity BEH C18, #186005225, Waters, Waters, Elstree, Herts, UK) using a running buffer consisting of 50 mM Tris and 150 mM NaCl, at pH 7.5. Absorbance was measured at 415 nm, the area under the curve was quantified and Hb concentration was calculated using the standard curve. Each assay run was quality controlled using three in-house Hp standards (200 μg/ml, 10 μg/ml and 1 μg/ml) covering the assay range.

Control individuals

Control participants were patients who underwent lumbar puncture for neurological symptoms or conditions which were non-inflammatory and non-haemorrhagic (e.g. headache, normal pressure hydrocephalus and idiopathic intracranial hypertension) and were confirmed to have normal CSF. The National Research Ethics Committee approval number was 11/SC/0204.

Measurement of CSF neurofilament light chain

NFL was measured by enzyme-linked immunosorbent assay (UmanDiagnostics, Umea, Sweden), according to the manufacturer's instructions. Briefly, samples were thawed at 21°C, and centrifuged at 1750 rcf for 5 minutes at 21°C. Samples were then diluted 1:2 with sample diluent and added in duplicate to microplate wells coated with a monoclonal capture antibody specific for NFL. Next, samples were incubated with a biotinylated NFL-specific monoclonal detection antibody. The detection complex was completed with the addition of horseradish peroxidase-labelled streptavidin and tetramethylbenzidine (TMB) substrate. Samples were read at 450nm. Sample concentrations were extrapolated from a standard curve, fitted using a 4-parameter logistic algorithm. Intra-assay CVs were less than 10%.

Preparation of Hb from mouse RBCs

Blood was collected from C57BL/6 mice following administration of a terminal dose of pentobarbital. In a laminar flow hood, 5ml of blood was brought to 15ml using phosphate-buffered saline (PBS) (#20012-019, Thermo Fisher, Loughborough, UK) and centrifuged at 480g for 15 minutes. The supernatant was removed using aspiration, which included carefully removing the buffy coat. This centrifugation/resuspension/aspiration procedure was repeated three times until the supernatant was clear and the leukocyte layer fully depleted, leaving 2ml of RBC containing solution. 1.5ml of this solution was removed by pipetting from the bottom of the tube and brought to 6ml using sterile, distilled water (#UKF7114, Baxter, Newbury, Berkshire). Following gentle and brief mixing, RBC ghost membranes were pelleted via a

13,000g centrifugation for 30 minutes. The Hb containing supernatant was collected and brought to 0.9% NaCl using a 10X stock solution (made using sterile water and filtered through a 0.45µm filter). This final solution was then passed through a 0.45µm filter. Protein content was determined using the bicinchoninic acid assay (#23227, Thermo Fisher, Loughborough, UK). Hb constitutes 98% of total RBC cytosolic protein (Liumbruno *et al.*, 2010; Lutz and Bogdanova, 2013), and the purity of each preparation was assessed by sodium dodecyl sulphate polyacrylamide gel electrophoresis and Coomassie staining (Supplementary Figure 7).

Primary neuronal cultures

Primary hippocampal neurones were cultured according to a modified method from the Ittner lab (Fath *et al.*, 2009; Garland *et al.*, 2012). This method utilizes a support ring of cortical cells in the periphery of a culture dish to provide trophic support to a low-density culture of hippocampal neurones on a central coverslip. Briefly, postnatal day 0 C57BL/6 mice were sacrificed and their hippocampal and cortical neurones dissociated using a kit designed for postnatal neurones (#130-094-802, Miltenyi, Bisley, Surrey, UK). Cortical and hippocampal cells were suspended in growth media (Neurobasal & 1X GlutaMAX, Thermo Fisher, Loughborough, UK; 1X Neurobrew, Miltenyi, Bisley, Surrey, UK) and plated at the following densities: 250,000/75µl cortical cells for the support ring, and 5000/50µl hippocampal cells per coverslip in 12 well plates. After four hours, suspension media was removed and replaced with 1 ml of pre-equilibrated growth media. Every 3–4 days 50% of culture media was replaced with fresh and pre-equilibrated growth media. Cultures were maintained at 5% CO_2 and 37°C, and used after seven days.

Immunocytochemistry

Cultured neurones were washed in PBS and fixed for 30 minutes with 4% paraformaldehyde (PFA). Following two five-minute washes with PBS, neurones were incubated at 21°C with blocking buffer (BB) (made of 5% normal goat serum, 1% BSA and 0.2% Triton X-100 in PBS), and then incubated with anti-MAP2 (1:500; #AB5622, Millipore, Merck, Watford, Hertfordshire, UK) overnight at 4°C in BB. Wells were washed with PBS three times, for five minutes each wash, and incubated with anti-rabbit Alexa488 (Thermo Fisher, Loughborough, UK) in PBS for one hour. Wells were then washed with PBS three times, for five minutes each wash. Coverslips were mounted onto glass slides using Prolong Gold with DAPI (#P36941, Thermo Fisher, Loughborough, UK).

Hb challenge and Hp treatment

Cultured neurones were challenged with Hb at day 7 until day 14. Over the 7 days, Hb concentration was maintained during media changes. An equimolar-binding ratio of Hp was added concomitantly for Hp treatment experiments.

Counting viable neurons

Following blinding, coverslips were imaged using a Leica DM5000 microscope under x40 magnification. The objective was passed over the full length of each coverslip and viable neurones identified as MAP2-positive with non-apoptotic nuclei. The mean from 2 coverslips for each repeat was taken.

Source of haptoglobin

Hp was purified from pooled human plasma at Bio Products Laboratory Limited (http://www.bplgroup.com), with a methodology (Dalton, 2012) that enriches for Hp dimer (Lipiski *et al.*, 2013) (dimer, 68%; trimer, 16%, higher polymeric forms, 16%); it was 98% pure as determined by UPLC. The preparation used in this study was a clinical grade formulation with <0.02 endotoxin units/ml. The Hb-binding capacity of this Hp preparation was determined empirically by incubating increasing concentrations of mouse Hb with a fixed concentration of Hp (1mg/ml), followed by UPLC quantification (Supplementary Figure 8). Binding was observed to saturate at a Hb to Hp mass ratio of 1:1.4.

Mouse model of prolonged intrathecal Hb exposure

To model prolonged intrathecal Hb exposure, a two-week intracerebroventricular infusion of Hb was used. Locally bred male C57BL/6 mice were housed at 21°C under a 12-hour light/12-hour dark cycle in a conventional animal research facility, and allowed access to food and water *ad libitum*; surgery was performed at 10-12 weeks of age. All procedures were performed under U.K. Home Office licence 30-3057 and all applicable international, national, and/or institutional guidelines for the care and use of animals were followed. Alzet osmotic minipumps (Model 2002, #296, two-week infusion, 0.5µl/hr, Charles River, Harlow, Essex) were loaded with vehicle (0.9% saline), Hb, Hb + Hp, or Hp. Treatment allocation was random. The infusate Hb concentration was 20mg/ml. To achieve a clinically relevant CSF Hb concentration of 10µM, the following parameters were used to guide optimization of the infusate Hb concentration: CSF volume in mice = 40µL (Rudick *et al.*, 1982); CSF formation rate = 0.33µL/min (Rudick *et al.*, 1982); C57BL/6J mouse brain weight = 0.45g (Rosen and Williams, 2001); interstitial fluid (ISF) is 20% of brain weight (Davson and Segal, 1996); rate

of ISF formation 0.17µL/g/min in rodents (Cserr *et al.*, 1981; Abbott and Bradbury, 1992). The Hp concentration was 14mg/ml to achieve 1:1 Hb to Hp binding stoichiometry, guided by data from empirical determination of Hb to BPL Hp binding ratio (Supplementary Figure 8). The minipump was attached to a 30-gauge ventricular cannula using vinyl tubing (Brain Infusion Kit 3, #8851, Charles River, Harlow, Essex), and primed with test solution according to the manufacturer's instructions. Mice were anaesthetised using a ketamine (Bayer, Reading, UK) and xylazine (Pfizer, Tadworth, Surrey, UK) mixture (100 and 10 mg/kg, respectively). The head was immobilized in a stereotactic frame. Mice body temperature was thermostatically controlled with a rectal probe and a heated pad. An aseptic technique was used. Blunt dissection was used to create a subcutaneous pocket in the interscapular region for insertion of the minipump. A burr hole was drilled and the ventricular cannula inserted at the following coordinates from bregma: anteroposterior, -0.4mm; lateral, 1mm; depth, 2.5mm. Cyanoacrylate was used to secure the cannula holder to the skull surface. Buprenorphine (Centaur, Castle Cary, Somerset, UK) was administered immediately following surgery (0.5mg/kg subcutaneously) and the next morning in 5ml fruit jelly (300µg/45ml). Mice were housed individually following surgery for 14 days so the experimental unit was a single animal. Mice were checked twice in the first 24 hours and daily thereafter. Residual volume in the minipumps was systematically examined after explantation, at the end of the two weeks, to ensure the appropriate volume of 200µL was delivered while *in situ*. Correct placement of the ventricular cannula was confirmed during tissue sectioning by observing the cannula track.

Open-field behavioural analysis

Open-field behavioural analysis was performed in a laser-monitored behaviour arena (Med Associates, Fairfax, VA, US) over a five-minute period, twice a day at the same time in the morning and afternoon. An ambulation score was calculated by combining the percentage change from baseline for three ambulatory readouts: distance travelled, ambulatory counts, and time spent ambulating. A freezing score was calculated by deriving the percentage change from baseline for the time spent in the outer 25% of the open field over the first minute (Supplementary Figure 9).

Immunohistochemistry

At 14 days post-surgery, mice were transcardially perfused with heparinised saline (1unit/ml, LEO Pharma, Hurley, Berkshire, UK), followed by 4% PFA in 0.1M phosphate buffer. Brains

were removed and post-fixed in 4% PFA overnight at 4°C and then embedded in paraffin wax. Coronal sections were cut at 10µm, dewaxed in xylene, and rehydrated through graded ethanol/water solutions. Protocols for each marker were as follows:

GFAP/Iba1

For antigen retrieval, sections were immersed in 10mM citrate buffer (pH6) and boiled in a microwave for 6 minutes. Endogenous peroxide was quenched using 0.3% v/v hydrogen peroxide in 1% v/v methanol for 30 minutes. Sections were blocked in 5% v/v normal goat serum + 0.1% w/v BSA for one hour at RT. In the same blocking solution, sections were incubated at 4°C overnight with primary antibody (1:500 anti-Iba1, Wako, Osaka, Japan; 1:2000 anti-GFAP, Dako, Agilent, Santa Clara, CA, US). Following three five-minute washes in PBS with 0.1% Tween-20 (PBS-T), sections were incubated with biotinylated anti-rabbit antibody (1:100, #BA-1000, Vector labs, Burlingame, CA, US) for one hour at RT in PBS. Sections were washed in PBS-T three times, for five minutes each wash, and then incubated in ABC solution (#PK-6100, Vector Labs, Burlingame, CA, US) for 45 minutes at RT. Sections were washed in PBS three times, for five minutes each wash, followed by development in 0.1M phosphate buffer containing 0.05% w/v 3,3'-Diaminobenzidine and 0.015% v/v hydrogen peroxide. Sections were counterstained in haematoxylin, dehydrated through graded ethanol/water solutions, cleared in xylene, and mounted using DPX.

Synaptophysin

For antigen retrieval, sections were incubated at 65°C in 0.2M boric acid (pH9) for 30 minutes. Endogenous peroxide was quenched using 0.3% hydrogen peroxide in PBS. Sections were blocked in 10% normal goat serum for one hour at RT in PBS and then incubated with anti-synaptophysin (1:2000, UK #5258-I, Millipore, Merck, Watford, Hertfordshire) in the same blocking solution overnight at 4°C. Sections were washed in PBS with PBS three times, for five minutes each wash, and then incubated with biotinylated anti-mouse antibody (#9200, Vector Labs, Burlingame, CA, US) in PBS for one hour at RT. Sections were washed in PBS three times, for five minutes each wash, and incubated with ABC solution for 45 minutes. Finally, sections were washed in PBS three times, for five minutes each wash, followed by development in 0.1M phosphate buffer containing 0.05% w/v 3,3'-Diaminobenzidine, 0.015% v/v hydrogen peroxide, and 0.06% w/v ammonium nickel sulphate. Sections were dehydrated through graded ethanol solutions, cleared in xylene, and mounted using DPX without counterstaining.

Haemoglobin/CD163

Sections were boiled in a citrate buffer (10mM, pH6) for two five-minute heat/cool cycles, washed twice for five minutes in PBS, and quenched in 3% hydrogen peroxide for 10 minutes in PBS. After two five-minute washes in PBS, sections were blocked with 10% goat serum + 1% BSA in PBS for one hour at RT. Blocking buffer was tapped off, and sections incubated in 1/500 anti-Hb (ab102758, Abcam, Cambridge, UK) or 1/500 anti-CD163 (ab182422, Abcam, Cambridge, UK) overnight at 4°C. Sections were washed in PBS three times, for five minutes each wash, and subjected to the DAB method as per section for 'GFAP/Iba1'. Both unstained and counterstained sections were prepared prior to mounting in DPX.

Smooth muscle actin

Fixed-frozen sections were warmed for 15min at 37°C and then blocked in 10% normal goat serum for 30min in PBS. Blocking buffer was tapped off and sections incubated with 1/100 anti-SMA-FITC (F377, Sigma-Aldrich, Gillingham, Dorset, UK) overnight in PBS+0.1% triton X-100 at 4°C. Sections were washed in PBS three times, for five minutes each wash, and mounted using Prolong Gold antifade reagent with DAPI (P36941, Thermo Fisher, Loughborough, UK).

Fluorescent imaging of Circle of Willis in situ

The method for *in situ* imaging was established in the Zipfel group (Aum *et al.*, 2017). Mice were anaesthetised using a ketamine (Bayer, Reading, UK) and xylazine (Pfizer, Tadworth, Surrey, UK) mixture (100 and 10mg/kg, respectively) and transcardially perfused through the left ventricle with 10mM glucose-PBS followed by 20 mL 20µM 5-(6)-carboxy-X-rhodamine, succinimidyl ester (ROX SE, Sigma-Aldrich, Gillingham, Dorset, UK) dye in 10mM glucose-PBS prior to perfusions with 4% PFA (in PBS). All perfusions were performed with solutions at 21°C, at a constant pressure of 80mmHg ±2mmHg using a GE Druck DP1705. Animals were decapitated, the calvaria removed, and post-fixed in 4% PFA in the dark at 4°C for 24 hours. Brains were removed under a dissection microscope to preserve the basal arteries. Then, brains were placed *en bloc* on a glass coverslip and gently covered in PBS before placing on the stage of a confocal laser scanning microscope (SP8, Leica, Wetzlar, Germany). Vessels were imaged at 1024 x 1024 pixel resolution with four line averaging, 10x magnification and 0.75 zoom using an excitation laser wavelength of 561 nm and monitoring emittance between wavelengths of 572 –699 nm. Z stacks were generated through the entire vessel with spacing of 10µm. The z stack data were visualized in two dimensions using the maximum projection function. Photomicrographs were acquired of the Anterior Cerebral Artery (ACA) and Middle Cerebral

Artery (MCA). Measurements of vessel diameter were made in ImageJ (National Institute of Health, USA) using the line tool and measure function by drawing from one wall of the vessel to the other in a perpendicular direction. Measurements were made at the narrowest point across the first 1 mm of the vessel.

Perls' staining

Following de-waxing, slides were rehydrated through graded ethanol/water solutions and then incubated at 40°C in 7% w/v potassium ferrocyanide and 2% v/v hydrochloric acid mixed in a 1:1 ratio for 1hr. After a brief wash in distilled water, endogenous peroxidase activity was quenched in 0.3% v/v hydrogen peroxide in Tris-buffered saline for 30 minutes at RT. Sections were developed in 0.1M phosphate buffer containing 0.05% w/v 3,3'-Diaminobenzidine and 0.015% v/v hydrogen peroxide for seven minutes. Of the three sections per slide, only two were counterstained with haematoxylin; the unstained section was used for quantification of Perls' staining. Sections were dehydrated through graded ethanol/water solutions and cleared in xylene prior to mounting with DPX.

Imaging

To ensure blinding, all samples were coded prior to image capture and analysis. Images were captured using a Leica DM5000 microscope. Images were captured from the hemisphere ipsilateral to the cannula. All analyses were performed by two-dimensional quantification, averaged over two sections per animal within each area studied, and data is presented per mm^2, unless otherwise indicated.

GFAP/Iba1

Images were captured under x40 magnification. Two main sampling regions were selected to represent the cortex/thalamus and the hippocampus caudal to the Hb infusion site. Each region was sub-sampled in three areas in a standardized way (Supplementary Figure 10A). For the first region, two sample sites were in the cortex and one in the thalamus. For the hippocampal region, one central sample site was within the hippocampal molecular layer, in between CA1 and CA3 sample sites within the stratum radiatum adjacent to the pyramidal cell layer. Cells positive for each marker were counted from two sections from each location.

Synaptophysin

Brightfield images were taken at x10 magnification in order to image multiple strata of the ipsilateral hippocampus. Synaptophysin staining was quantified using the method established

by Cunningham *et al* (Cunningham *et al.*, 2003), to quantify staining as a ratio between regions normalised to the background staining intensity. Briefly, hippocampal strata pixel densities were measured using ImageJ software (National Institute of Health, USA) with measurements made at a light intensity within the linear transmittance (T) range. The dentate gyrus (DG) granular cell layer (the area of highest transmittance) was used as an internal control for each section and other strata transmittances were adjusted by subtraction of the DG transmittance from this layer's transmittance. A ratio was then calculated using the adjusted transmittances for the SR (the most variably stained regions) and the SLM (a consistently low stained region): Ratio = $(T_{DG} - T_{SR})/(T_{DG} - T_{SLM})$. Two sections per animal were analysed and a mean ratio calculated.

Smooth-muscle actin

Fluorescent images were captured under x10 magnification. Two sites either side of the ipsilateral lateral ventricle were used. Between 4-6 SMA+ vessels in a transverse orientation were imaged per animal resulting in ≈200 vessels being analysed across all groups. In ImageJ, each vessel was magnified and its lumen diameter and wall thickness measured. A ratio of lumen perimeter to wall thickness was calculated (Sabri and Macdonald, 2012). There was not a specific vessel size range; only vessels strictly within the brain tissue (i.e. not in the leptomeninges) were analysed.

Haemoglobin

Images of non-counterstained sections were collected under brightfield microscopy at x10 magnification. Three images were taken for each animal in the ipsilateral hemisphere. Image analysis in ImageJ used the same macro as per 'Perls' staining' below.

Perls' staining

Brightfield images of non-counterstained sections were obtained at x20 magnification; the convexity was sampled at the base of the brain and lateral aspect (Supplementary Figure 10B); the parenchyma was sampled either side of the lateral ventricle (Supplementary Figure 10A). Analysis was carried out using ImageJ. Following conversion to 8 bit format, all images were subjected to the same thresholding. The area fraction of staining was then quantified to represent the degree of non-haem iron deposition.

Western blotting

The hemisphere ipsilateral to cannula insertion was homogenised in RIPA buffer. 20µg of protein was separated on TGX 4-15% gels (BioRad, Watford, Hertfordshire, UK) and

transferred onto PVDF using a Trans-Blot Turbo system (BioRad, Watford, Hertfordshire, UK). The membrane was blocked in 3% BSA in Tris-buffed saline (pH 7.4) containing 0.1% Tween-20 (TBS-T) for one hour at RT. Membranes were incubated at 4ºC overnight in the same blocking buffer containing 1:1000 mouse anti-postsynaptic density protein 95 (#ab13552, Abcam, Cambridge, UK), and 1:1000 mouse anti-tubulin β3 (#801201, Biolegend, San Diego, CA, US). Membrane was washed in TBS-T three times, for five minutes each wash, and incubated with and Alexa546-conjugated anti-mouse IgG (Invitrogen, Carlsbad, CA, US) for one hour at RT. Following three five-minute washes in TBS-T, the membrane was imaged using a ChemiDoc system (BioRad, Watford, Hertfordshire, UK) with exposure set to prevent saturated pixels.

Surface plasmon resonance

SPR analysis was carried out as described (Madsen *et al.*, 2004) using a Biacore 3000 instrument (Biacore, Uppsala, Sweden). Mouse or human CD163 (produced recombinantly in house (Kristiansen *et al.*, 2001; Etzerodt *et al.*, 2013)) were immobilized in 10mM sodium acetate (pH 4) and remaining binding sites were blocked with 1M ethanolamine (pH 8.5). The resulting densities were: human CD163 0.0468 pmol/mm^2, and murine CD163 0.0320 pmol/mm^2. Sensorgrams were generated using the running buffer CaHBS with 2mM free Ca_2Cl_2 (10mM Hepes, 150mM NaCl, 3.0mM $CaCl_2$, 1.0mM EGTA, + 0.005% P20, and pH 7.4) and the protein concentrations: 5µg/ml of mouse and human Hb (Sigma-Aldrich, Gillingham, Dorset, UK); 7.5 µg/ml of mouse Hp (MyBioSource, San Diego, Ca) and human Hp1-1 (Sigma-Aldrich, Gillingham, Dorset, UK). The flow cell was regenerated in 10mM glycine, 20mM EDTA, 500mM NaCl+0.005% P20, pH4.0 between the runs. All experiments were at least conducted in triplicate and data was evaluated using the BiaEvaluation ver. 4.1 software (Biacore, Uppsala, Sweden).

Statistics

The normal reference range for NFL was derived from control individuals by using the natural log normal distribution. Values were natural log transformed and the upper and lower limits were calculated as three standard deviations on either side of the mean. Where possible, experiments were conducted using a 2x2 factorial design to reduce the number of animals used (Festing *et al.*, 2016) in line with the principle of 3Rs (Festing *et al.*, 2016). These experiments were analysed with a two-way analysis of variance (ANOVA) considering Hp and Hb as

factors with two levels each. Kruskal-Wallis test was used for non-parametric factorial data. The three primary hypotheses were: (1) Hb is toxic (2) Hp is not toxic (2) Hp protects against Hb toxicity. Mice received four types of intrathecal infusions: Hb only (n=16), Hb with an equimolar-binding ratio of Hp (n=15), Hp alone (n=9) or vehicle (n=13). *p* values were corrected for multiple comparisons using the false discovery rate method (Benjamini and Hochberg, 1995) with Q=0.05; sensitivity analyses using the full pairwise Bonferroni correction showed results were robust (Supplemental Table 2). One-way ANOVA was used to study the toxicity of different Hb concentrations *versus* control, for which multiple comparisons were corrected using Dunnett's. Weighted Pearson's r was used to determine correlations. A linear-mixed modelling approach was used to analyse time-series behavioural data, with a first order ante dependence covariance structure used to model correlated terms under heteroscedasticity. Within-subject random effects were modelled using random intercepts. Model comparison was assessed using Akaike information criterion and the log-likelihood ratio. Normality and heteroscedasticity was routinely determined across all data sets. Where necessary, logarithmic transformation was used to normalise data. α, the probability of a Type I error, was 0.05. Two-tailed hypotheses were considered throughout. Statistical analysis and graph preparation were performed using SPSS (v24) and GraphPad Prism (v7.01), with data expressed as mean ± standard error of the mean (SEM), median ± interquartile range, or 95% confidence intervals. Supplemental Table 2 details all the analyses performed and the results. Animals were randomly selected and sequentially assigned to treatment groups. Animal *in vivo* experiments were reported according to ARRIVE guidelines, and the checklist is available as Supplemental data (Kilkenny *et al.*, 2010).

Supplementary References

Abbott NJ, Bradbury MWB. Physiology and pharmacology of the blood-brain barrier. Handbook of experimental pharmacology. Berlin; New York: Springer-Verlag; 1992. p. 549.

Aum DJ, Vellimana AK, Singh I, Milner E, Nelson JW, Han BH, *et al.* A novel fluorescent imaging technique for assessment of cerebral vasospasm after experimental subarachnoid hemorrhage. Sci Rep 2017; 7(1): 9126.

Benjamini Y, Hochberg Y. Controlling the False Discovery Rate: A Practical and Powerful Approach to Multiple Testing. Journal of the Royal Statistical Society Series B (Methodological) 1995; 57(1): 289-300.

Cserr HF, Cooper DN, Suri PK, Patlak CS. Efflux of radiolabeled polyethylene glycols and albumin from rat brain. Am J Physiol 1981; 240(4): F319-28.

Cunningham C, Deacon R, Wells H, Boche D, Waters S, Diniz CP, *et al.* Synaptic changes characterize early behavioural signs in the ME7 model of murine prion disease. Eur J Neurosci 2003; 17(10): 2147-55.

Dalton JO, Azubuike. Haptoglobin. In: Bertolini JG, Neil; Curling, John, editor. Production of Plasma Proteins for Therapeutic Use. Hoboken, New Jersey: John Wiley & Sons, Inc.; 2012.

Davson H, Segal MB. Physiology of the CSF and blood-brain barriers. Boca Raton: CRC Press; 1996.

Etzerodt A, Kjolby M, Nielsen MJ, Maniecki M, Svendsen P, Moestrup SK. Plasma clearance of hemoglobin and haptoglobin in mice and effect of CD163 gene targeting disruption. Antioxidants & redox signaling 2013; 18(17): 2254-63.

Fath T, Ke YD, Gunning P, Gotz J, Ittner LM. Primary support cultures of hippocampal and substantia nigra neurons. Nat Protoc 2009; 4(1): 78-85.

Festing MFW, Overend P, Borja MC, Berdoy M. The design of animal experiments: reducing the use of animals in research through better experimental design. 2nd edition / Revised and updated edition. ed. Los Angeles: SAGE; 2016.

Galea J, Cruickshank G, Teeling JL, Boche D, Garland P, Perry VH, *et al.* The intrathecal CD163-haptoglobin-hemoglobin scavenging system in subarachnoid hemorrhage. J Neurochem 2012; 121(5): 785-92.

Garland P, Broom LJ, Quraishe S, Dalton PD, Skipp P, Newman TA, *et al.* Soluble axoplasm enriched from injured CNS axons reveals the early modulation of the actin cytoskeleton. PLoS One 2012; 7(10): e47552.

Kilkenny C, Browne WJ, Cuthill IC, Emerson M, Altman DG. Improving bioscience research reporting: the ARRIVE guidelines for reporting animal research. PLoS Biol 2010; 8(6): e1000412.

Kristiansen M, Graversen JH, Jacobsen C, Sonne O, Hoffman HJ, Law SK, *et al*. Identification of the haemoglobin scavenger receptor. Nature 2001; 409(6817): 198-201.

Lipiski M, Deuel JW, Baek JH, Engelsberger WR, Buehler PW, Schaer DJ. Human Hp1-1 and Hp2-2 phenotype-specific haptoglobin therapeutics are both effective in vitro and in guinea pigs to attenuate hemoglobin toxicity. Antioxidants & redox signaling 2013; 19(14): 1619-33.

Liumbruno G, D'Alessandro A, Grazzini G, Zolla L. Blood-related proteomics. J Proteomics 2010; 73(3): 483-507.

Lutz HU, Bogdanova A. Mechanisms tagging senescent red blood cells for clearance in healthy humans. Front Physiol 2013; 4: 387.

Madsen M, Moller HJ, Nielsen MJ, Jacobsen C, Graversen JH, van den Berg T, *et al*. Molecular characterization of the haptoglobin.hemoglobin receptor CD163. Ligand binding properties of the scavenger receptor cysteine-rich domain region. The Journal of biological chemistry 2004; 279(49): 51561-7.

Rosen GD, Williams RW. Complex trait analysis of the mouse striatum: independent QTLs modulate volume and neuron number. BMC Neurosci 2001; 2: 5.

Rudick RA, Zirretta DK, Herndon RM. Clearance of albumin from mouse subarachnoid space: a measure of CSF bulk flow. J Neurosci Methods 1982; 6(3): 253-9.

Sabri M, Macdonald RL. Vasospasm: Measurement of Diameter, Perimeter, and Wall Thickness. In: Chen J, Xu X-M, Xu ZC, Zhang JH, editors. Animal Models of Acute Neurological Injuries II: Injury and Mechanistic Assessments, Volume 1. Totowa, NJ: Humana Press; 2012. p. 473-9.

Supplementary Table 1. Statistical methods and results.

Figure		n number: Control/Normal	n number: SAH	Test	Result	p-value
2a	Maximum NFL	19	42	unpaired T-test	t=8.504,df=59	<0.0001
2c	Normal vs High NFL	12	30	unpaired T-test	t=2.757,df=40	0.0087
2d	Ln Total Hb vs Ln predicted_NFL	N/A	42	Multivariable linear regression	B=0.364(0.156-0.573)	0.001

	Figure		n number				Test		Result	p-value	Group comparisons: false discovery rate, Q=0.05			Group comparisons: Bonferroni			Group comparisons: other		
			Vehicle	Hb	Hb+Hp	Hp					Vehicle vs Hb	Vehicle vs Hp	Hb vs Hb+Hp	Vehicle vs Hb	Vehicle vs Hp	Hb vs Hb+Hp	Vehicle vs Hb	Vehicle vs Hp	Hb vs Hb+Hp
	3c	*in vitro* Hb titration	5	5(all conditions)	N/A	N/A	One-way ANOVA		F(5,24)=17.89	<0.0001							0.001 (10μM, D1)		
	3d	*in vitro* Hp efficacy	5	5	5	5	two-way ANOVA	interaction	F(1,16)=11.98	0.0032	0.003	ns	0.0016	0.0059	ns	0.0016			
	4f	post-surgery ambulation	11	16	15	9	LMM	interaction	F(1,39.229)=4.136	0.049							0.003	ns	0.036
	4g	post-surgery freezing	11	16	15	9	LMM	Interaction	F(1,45.621)=3.572	0.065							0.025	ns	0.041
	5e	cortex Iba1+ IHC	13	11	10	7	two-way ANOVA	interaction	F(1,37)=10.13	0.003	0.0002	ns	0.0156	0.000198	ns	0.0312			
	5i	SR+ Iba1+ IHC	11	11	10	7	two-way ANOVA	interaction	F(1,35)=1.364	0.2508	0.0494	ns	0.0494	ns	ns	ns			
								main effect_Hb	F(1,35)=4.455	0.042									
								main effect_Hp	F(1,35)=2.889	0.0981									
	5m	SR+ GFAP+ IHC	13	11	9	8	two-way ANOVA	interaction	F(1,37)=5.376	0.026	0.0164	ns	0.0199	0.0164	ns	0.0397			
	5q	hippocampal SY38 IHC	11	10	10	8	two-way ANOVA	interaction	F(1,35)=3.481	0.0705	0.0028	0.0018	0.0028	0.0084	0.0018	0.0066			
								main effect_Hb	F(1,35)=8.055	0.0075									
								main effect_Hp	F(1,35)=9.392	0.0042									
	6a	CD163 IHC	8-13	10-12	9-10	7-8	Kruskal-Wallis			ns	ns	ns	ns				ns	ns	ns
	6d	Perls' parenchyma	12	11	9	8	Kruskal-Wallis			0.0002	0.0004	ns	0.0385				0.0004(D2)	ns	ns
	6e	Perls' convexity	13	12	9	8	Kruskal-Wallis			0.0003	0.0008	ns	ns				0.0011(D2)	ns	ns
	7c	Ipsilateral MCA width	11	11	11	11	two-way ANOVA	interaction	F(1,40)=1.020	0.3186	0.0249	ns	ns	0.0498	ns	0.0128			
								main effect_Hb	F (1,40)=8.514	0.0058									
								main effect_Hp	F(1,40)=2.986	0.0917									
	7d	Ipsilateral parenchymal SMA+ vessels	11	11	11	11	two-way ANOVA	interaction	F(1,40)=6.062	0.0182	0.0296	ns	0.0171	0.0593	ns	0.0171			
	7e	Ipsilateral parenchymal Hb IHC	11	11	10	9	Kruskal-Wallis			0.0001	0.0249	ns	0.0013				ns	ns	0.0076(D2)
	S2	Ambulation scores AUC	7	12	10	5	two-way ANOVA	interaction	F(1,30)=4.097	0.0519	0.0206	ns	ns	0.0034	ns	0.0176			
								main effect_Hb	F(1,30)=0.8865	0.3539									
								main effect_Hp	F (1,30)=4.589	0.0404									
	S3a	total hippocampus Iba1+ cells	11	11	10	7	two-way ANOVA	interaction	F(1,36)=0.06971	0.7933	ns	ns	ns	ns	ns	ns			
								main effect_Hb	F(1,36)=2.872	0.098									
								main effect_Hp	F (1,36)=0.5818	0.4506									
	S3b	total hippocampal GFAP+ cells	13	11	9	8	two-way ANOVA	interaction	F(1,37)=1.082	0.3051	0.0282	ns	0.0499	0.0564	ns	ns			
								main effect_Hb	F(1,37)=6.208	0.0173									
								main effect_Hp	F(1,37)=5.135	0.0294									
	S3c	cortex GFAP+ cells	5	6	7	4	two-way ANOVA	interaction	F(1,17)=0.2886	0.5981	ns	ns	ns	ns	ns	ns			
								main effect_Hb	F(1,17)=0.02637	0.8729									
								main effect_Hp	F(1,17)=0.4274	0.522									
	S4	PSD-95 immunoblot	4	4	4	4	two-way ANOVA	interaction	F(1,12)=3.689	0.0788	0.005	ns	0.005	0.0106	ns	0.0149			
								main effect_Hb	F(1,12)=13.92	0.0029									
								main effect_Hp	F(1,12)=12.02	0.0047									
Parenchyma	S5a	Perls vs cortical Iba1+ cells	13	11	9	7	weighted Pearson's r		0.9	0.05									
Parenchyma	S5b	Perls vs total hippocampal Iba1+ cells	11	12	9	8	weighted Pearson's r		0.976	0.012									
Parenchyma	S5c	Perls vs total hippocampal GFAP+ cells	13	11	8	8	weighted Pearson's r		0.934	0.033									
Parenchyma	S5d	Perls vs hippocampal SY38 IHC	11	10	9	8	weighted Pearson's r		-0.961	0.02									
Parenchyma	S5e	Perls vs AUC ambulation	7	12	9	5	weighted Pearson's r		-0.996	0.002									
Parenchyma	S5f	Perls vs AUC freezing	7	12	9	5	weighted Pearson's r		0.932	0.034									
Convexity	S5g	Perls vs cortical Iba1+ cells	12	10	9	7	weighted Pearson's r		0.898	0.051									
Convexity	S5h	Perls vs total hippocampal Iba1+ cells	11	11	9	8	weighted Pearson's r		0.939	0.031									
Convexity	S5i	Perls vs total hippocampal GFAP+ cells	12	10	8	8	weighted Pearson's r		0.956	0.022									
Convexity	S5j	Perls vs hippocampal SY38 IHC	10	9	9	8	weighted Pearson's r		-0.985	0.008									
Convexity	S5k	Perls vs AUC ambulation	7	11	9	5	weighted Pearson's r		-0.961	0.039									
Convexity	S5l	Perls vs AUC freezing	7	11	9	5	weighted Pearson's r		0.883	0.059									
	S6a	Ipsilateral ACA width	11	10	11	10	two-way ANOVA	main effect Hb	F(1,38)=3.444	0.0712	ns	ns	ns	ns	ns	ns			
	S6b	Contralateral ACA width	11	10	11	9	two-way ANOVA	main effect Hb	F(1,37)=8.319	0.0065	0.0618	ns	ns	ns	ns	ns			
	S6c	Contralateral MCA width	11	11	10	11	two-way ANOVA	main effect Hb	F(1,39)=6.440	0.0153	ns	ns	ns	ns	ns	ns			

Note: in two-way ANOVAs, main effects (Hb and Hp) are only shown in the absence of an interaction (Hb x Hp) with p <0.05

Key:
D1=Dunnett's
D2=Dunn's
ns=non-significant
LMM=linear mixed modelling
significant test
significant p-value
marginal p-value

Supplementary Figure 1

Free iron assay. Mouse Hb is stable following a two-week incubation at 37°C, with negligible release of free chelatable iron. 50µM mouse Hb ± an equimolar-binding ratio of Hp was incubated for 0, 7 and 14 days. After reduction to its ferrous state, free iron was analysed via reaction with the chelator TPTZ [2,4,6-Tri-(2-pyridyl)-5-triazine]; absorbance was measured at 600/800nm on an AU5800 Beckman Coulter analyser. n=6 experiments each with duplicate assays per condition, mean ±SEM.

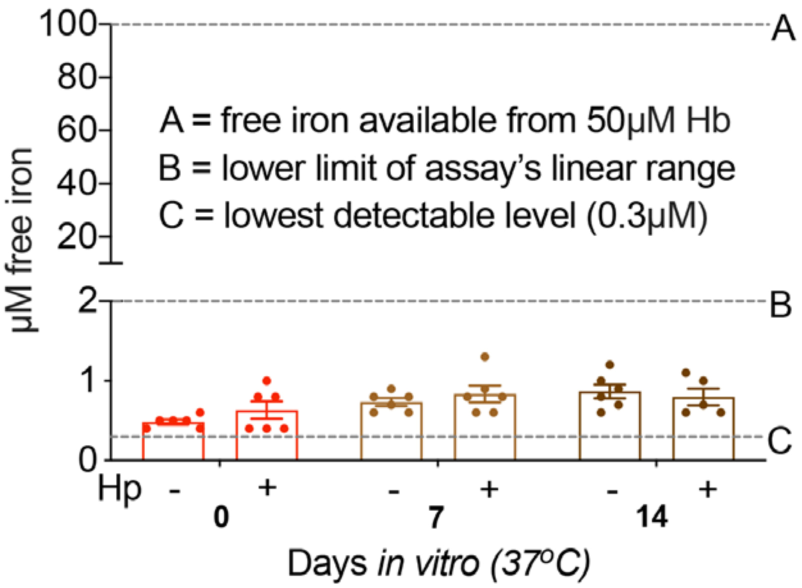

Supplementary Figure 2

Ambulation scores during laser-monitored open-field behavioural testing, not normalized to baseline (n=9-16 per condition). Mean area under the curve ±SEM. p values $*p < 0.05$, $**p < 0.01$.

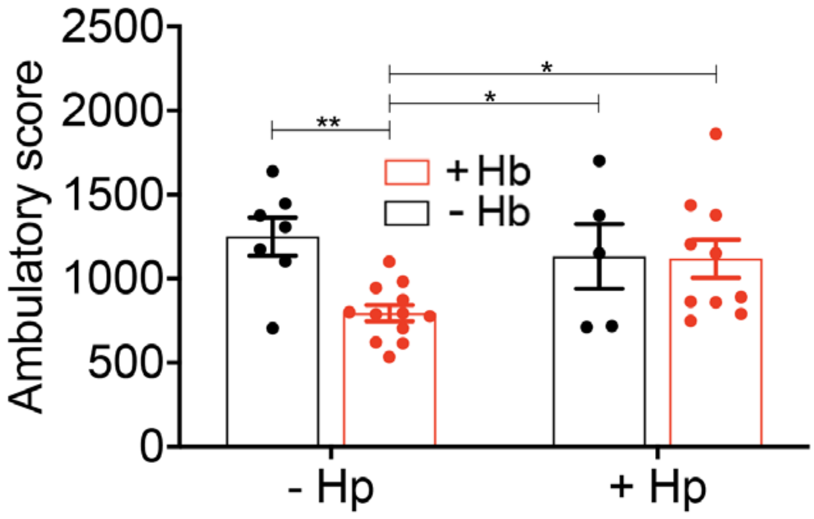

Supplementary Figure 3

Further Iba1 and GFAP immunohistochemistry. Quantification of total hippocampus lba1+ cell numbers ipsilateral to Hb infusion (**a,** n=7-11 per condition). Quantification of GFAP+ cell numbers ipsilateral to Hb infusion in total hippocampus (**b**, n=8-13 per condition) and cortex (**c**, n=7-10 per condition). Mean ±SEM. *p* values **p* < 0.05.

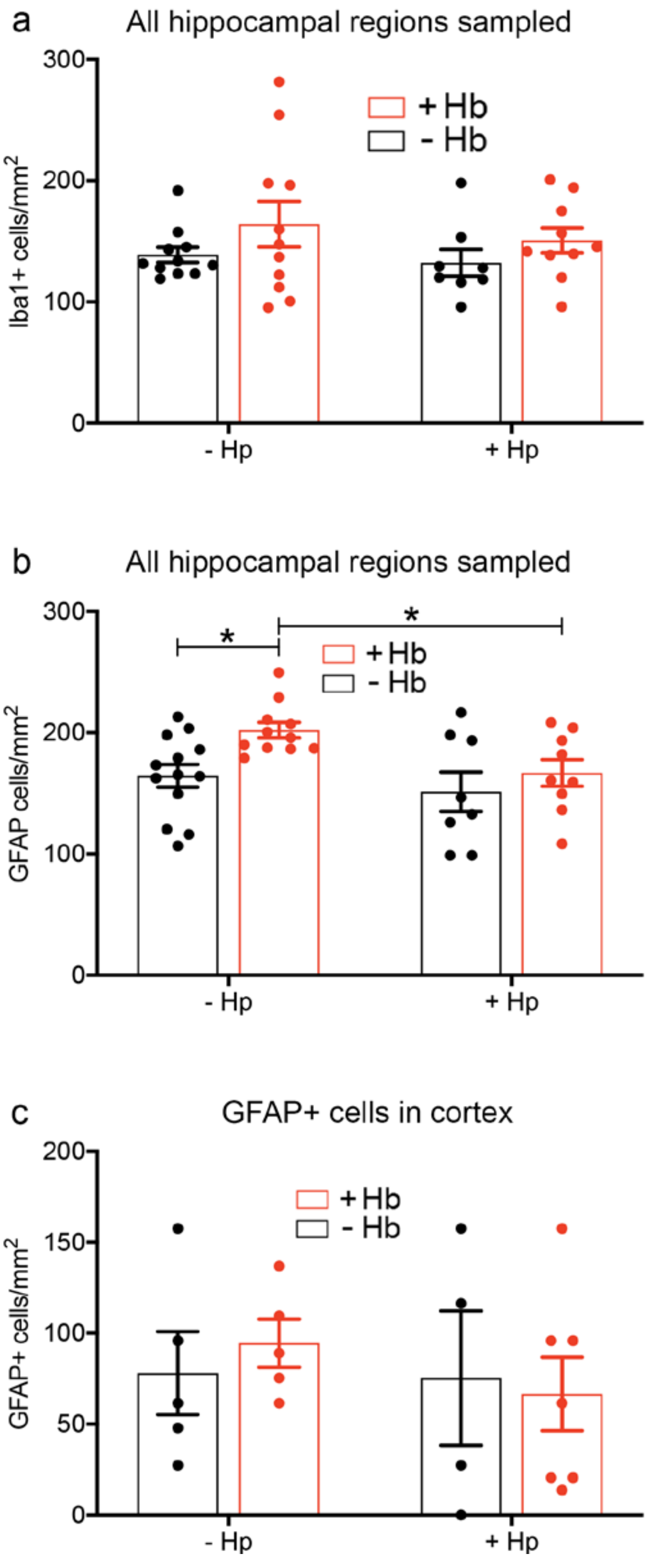

Supplementary Figure 4

PSD95 western blot. Immunoblot for postsynaptic density protein 95 (psd-95) in the hemisphere ipsilateral to Hb infusion (n=4 per condition, mean ±SEM). p values $^{**}p < 0.01$. Cropped and full length versions of a representative blot are shown.

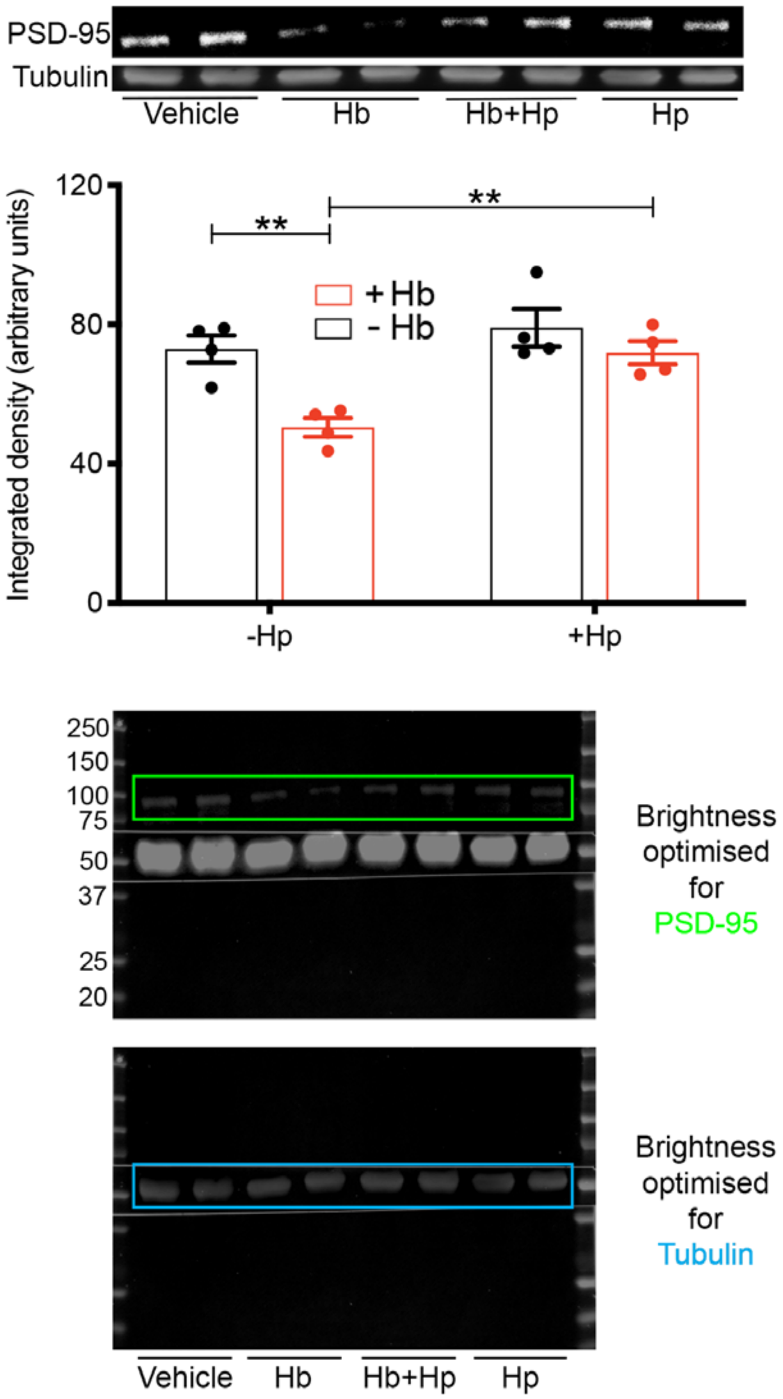

Supplementary Figure 5

Correlations of Perls' staining with pathology. Iron deposition is correlated with markers of inflammation, injury, synapse loss, and behavioural deficits. Animals are grouped by treatment representing high (Hb), low (vehicle & Hp), and intermediate (Hb+Hp) iron deposition. Weighted Pearson's correlations were calculated between Perls' staining (**a**,**b**,**c**,**d**,**e**,**f**, parenchyma; **g**,**h**,**i**,**j**,**k**,**l**, convexity) and immunohistochemical (n=7-13 per condition, mean ±SEM) and behavioural readouts (n=5-12 per condition, mean ±SEM). Perls' staining correlated positively with Iba1+ cells in the cortex (**a**,**g**), Iba1+ cells in the whole hippocampus (**b**,**h**), GFAP+ cells in the whole hippocampus (**c**,**i**), and freezing behaviour (**f**,**l**). Perls' staining correlated negatively with hippocampal Sy38 (**d**,**j**) and ambulation (**e**,**k**).

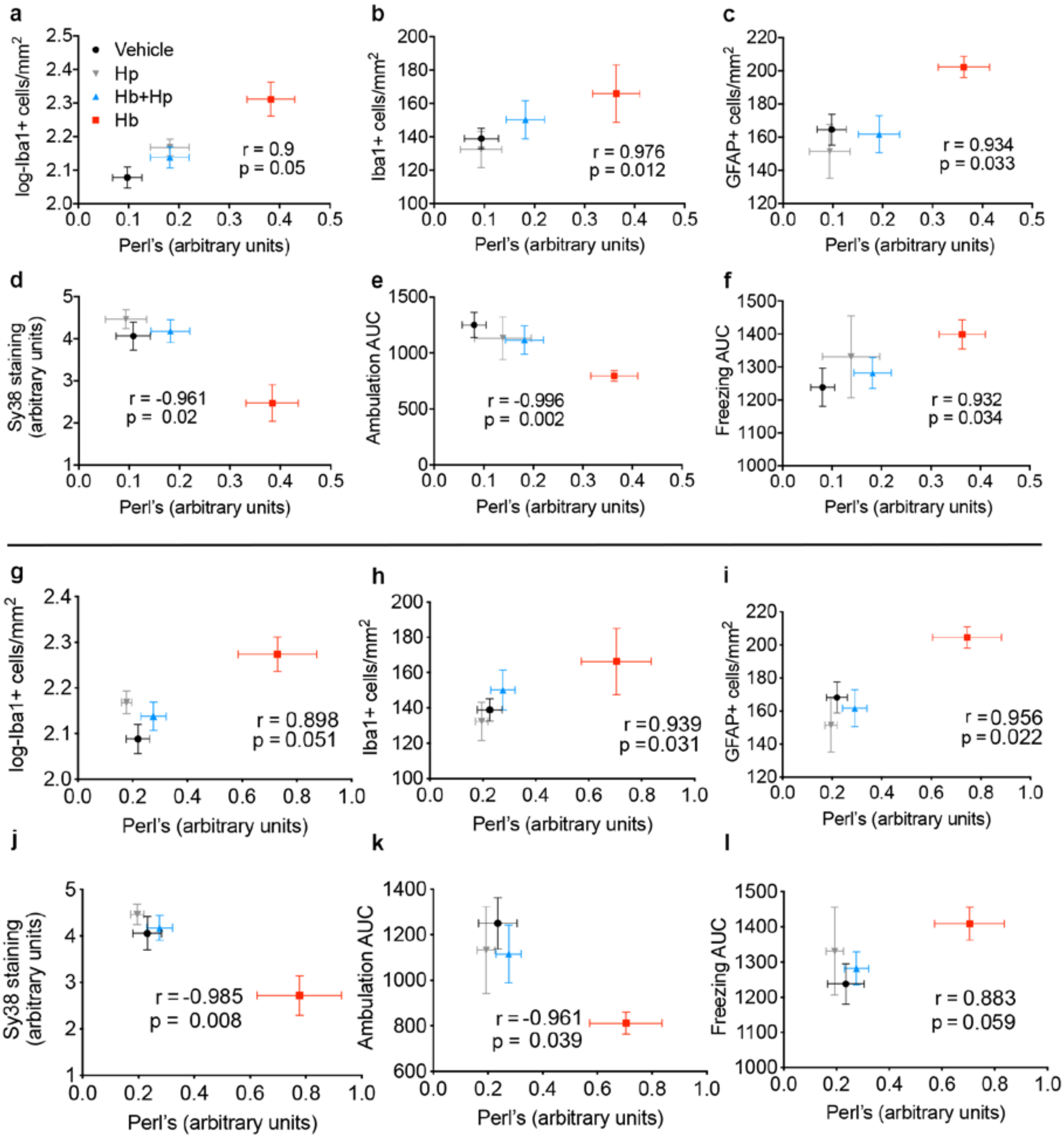

Supplementary Figure 6

Large cerebral artery vasospasm. Quantification of arterial diameter of cerebral arteries, other than the ipsilateral MCA. **a**, Ipsilateral anterior cerebral artery (ACA; n=10-11, two-way ANOVA: main effect Hb=0.0712, mean ±SEM). **b**, Contralateral ACA (n=9-11, two-way ANOVA: main effect Hb=0.0065, mean ±SEM). **c**, Contralateral MCA (n=10-11, two-way ANOVA: main effect Hb=0.0153, mean ±SEM). *p* value $*p < 0.05$.

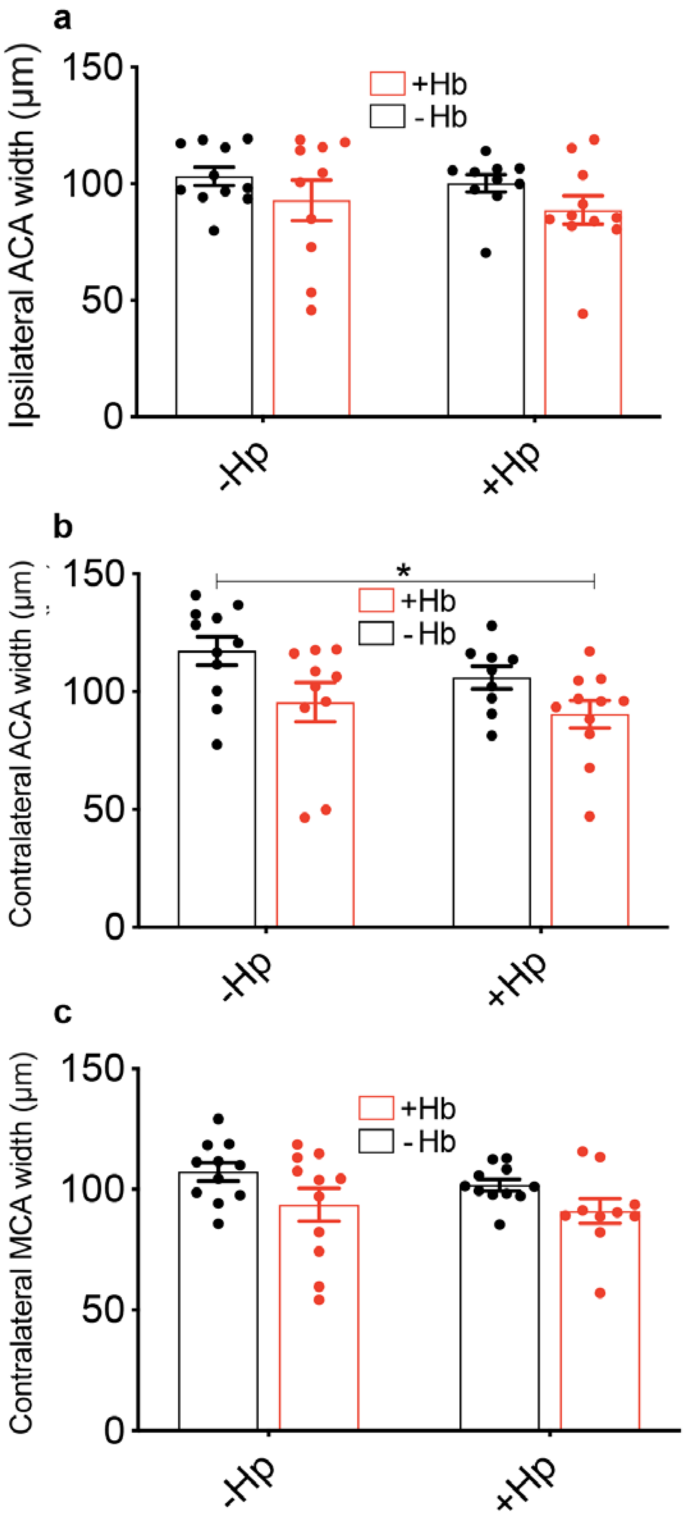

Supplementary Figure 7

Purity of mouse Hb preparation. SDS-PAGE of multiple Hb preparations demonstrates purity of Hb preparation. 10μg of total protein from seven individual Hb preparations were separated using reducing SDS-PAGE. Coomassie stain was used to identify protein bands. Only one protein (Hb) was visible.

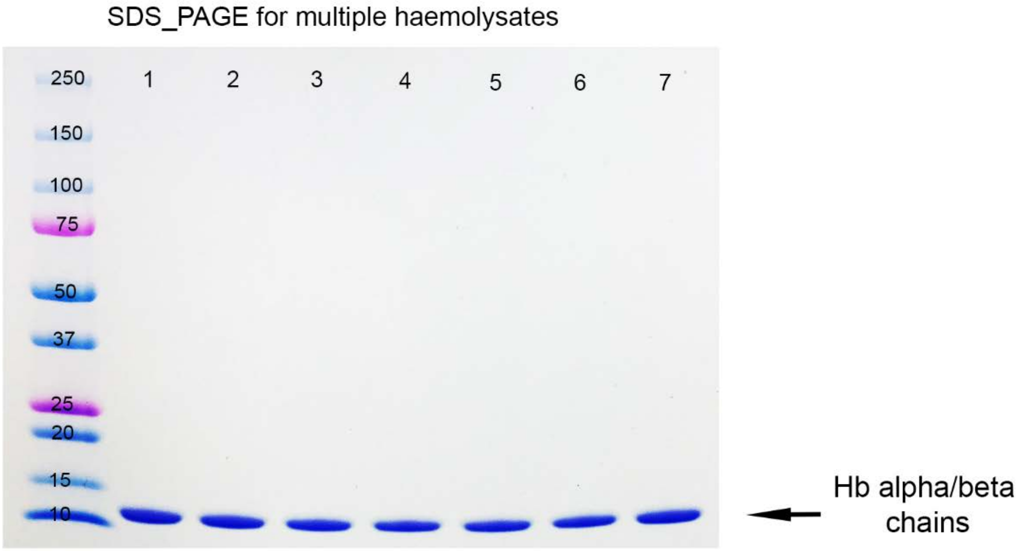

Supplementary Figure 8

Hb-Hp binding curve. Determination of Hb to Hp binding ratio. Increasing concentrations of mouse Hb were incubated with a fixed concentration of human Hp (1 mg/ml) and analysed using UPLC (see Methods). n=3, ±SEM. Saturated binding was observed at a 1:1.4 Hp to Hb ratio, identical to the binding ratio of the same batch of haptoglobin to human haemoglobin.

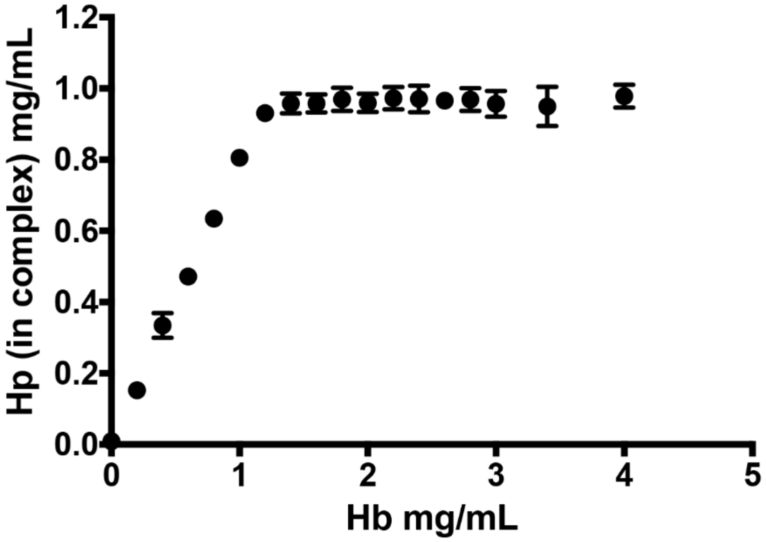

Supplementary Figure 9

Freezing score. Zonal analysis of open-field data. To assess acute freezing behaviour, the open-field arena was partitioned into inner and outer zones and the amount of time spent immobile in the outer zone recorded in the first minute.

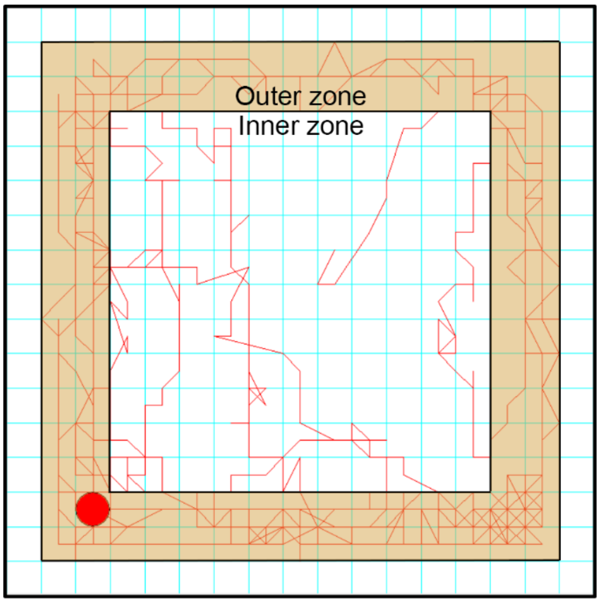

Supplementary Figure 10

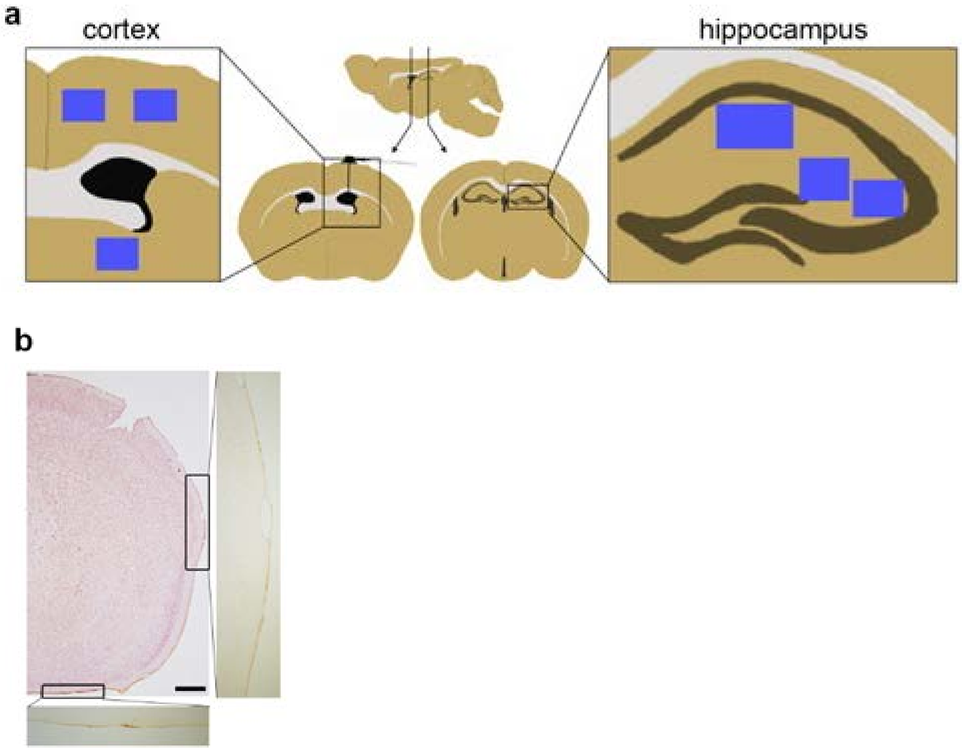

Supplement: fcz053_Supplementary_Data [file fcz053_supplementary_data.pdf]
